# An ethnobotanical survey of medicinal plants in Trinidad

**DOI:** 10.1186/s13002-015-0052-0

**Published:** 2015-09-15

**Authors:** Y. N. Clement, Y. S. Baksh-Comeau, C. E. Seaforth

**Affiliations:** Department of Paraclinical Sciences, The University of the West Indies, St. Augustine, Trinidad and Tobago; Department of Life Sciences, The University of the West Indies, St. Augustine, Trinidad and Tobago; Herbal Institute, The University of Trinidad and Tobago, St. Augustine, Trinidad and Tobago

**Keywords:** ‘Cooling/cleanser’, ‘Stoppage of water’, ‘Afterbirth’, ‘Womb infection’, Kidney stones, *Leonotis nepetifolia*, *Gomphrena globosa*, *Senna occidentalis*, *Cymbopogon citratus*

## Abstract

**Background:**

An ethnobotanical survey was conducted on the Caribbean island of Trinidad to identify medicinal plants commonly used in traditional medicine to treat a variety of medical conditions.

**Methods:**

A pilot survey was conducted to identify the top ten most common ailments where medicinal plants were used. The results of the foregoing study guided a wider national survey conducted between October 2007 and July 2008. A total of 450 households from 50 rural communities were interviewed using the TRAMIL (Traditional Medicine in the Islands) questionnaire for data collection. Details of plants, part(s) used, and remedy formulations were elicited from informants and voucher specimens collected for identification at the National Herbarium of Trinidad and Tobago. The TRAMIL methodology set a limit of a plant with 20 % or more citations for any particular ailment as having significant or popular use.

**Results:**

At the end of the survey 917 single plant remedies were identified. The majority of species were from the following families; Asteraceae, Lamiaceae, Leguminosae, Verbenaceae and Poaceae. Applying the TRAMIL 20 % citation of a plant for popular use as significant, *Leonotis nepetifolia (*for cough/common cold), *Gomphrena globosa* (for “stoppage-of-water”), *Curcuma longa* and *Senna occidentalis* (for “afterbirth”), *Cymbopogon citratus* and *Neurolaena lobata (*for fever), and *Citrus limon* (for kidney stones) qualified in our study. Those not reaching the TRAMIL 20 % significant (popular) use were *Stachytarpheta jamaicensis* (L.) Vahl*, Senna alata* (L.) Roxb.and *Momordica charantia* L. which were widely used as “‘cooling/cleanser’” in our survey.

**Conclusions:**

Our survey showed significant retention of traditional knowledge of medicinal plants in rural Trinidad. More interestingly, a large remnant of medico-cultural concepts such as “cooling/cleanser”, “afterbirth”, “stoppage-of-water” and “womb infection” persist in the rural population. Although the scientific literature show that some of the cited plants possessed antimicrobial, anti-inflammatory and related pharmacological activities in laboratory studies, these results must be taken with caution until clinical trials are conducted to establish safety and efficacy.

## Background

The island of Trinidad which lies approximately 13 km off the coast of the Paria Peninsula of Venezuela is the larger of the twin-island state of the Republic of Trinidad and Tobago. The island has a population of approximately 1.3 million people with about 77 % being either of African or Asian Indian ancestry or an admixture of these major ethnic groups [1]. Unlike other Caribbean islands, Trinidad is a continental island sharing its geology, flora and fauna, with South America having recently separated from the mainland ca. 10,000 years ago [2]. This gives Trinidad a unique mix of Antillean and South American elements in its flora and fauna. However, the natural vegetation has been significantly transformed in the post-Columbian era with the arrival of the Europeans, West Africans and the East Indians [3]. Today, approximately one-third of the flora is made up of exotic species which are fully incooperated into the modern herbal medicine repertoire, for example, ginger (*Zingiber officinale* Roscoe) (Baksh-Comeau YS, Maharaj SS, Harris SA, Filer DL, Hawthorne WD: An annotated checklist to the vascular plants of Trinidad and Tobago, unpublished).

The Caribbean region has a long history of using herbal medicine for disease management and maintenance of health. The native Amerindians incorporated indigenous ]species of medicinal plants in their rituals as part of their healthcare system. These First Nation people were gradually replaced by the sequential arrivals of European settlers, enslaved Africans, indentured Asian Indians and other minority ethnic groups. By and large the peoples who came to the region brought with them inherent knowledge of the use of medicinal plants, substituting with the local flora, which over time has led to the development of herbal pharmacopeias in the region [4–6]. There has been a significant loss of this folkloric knowledge, which depended on the oral tradition, for its transmission to successive generations. The main causes of this loss were due to migration, urbanization, modernization and the acceptance of western medicine strongly rooted over the last century. Therefore it is imperative that a concerted effort be made to document and preserve this residual knowledge [7].

Earlier ethnobotanical surveys in Trinidad were mostly qualitative in nature, and restricted in scope with regard to health conditions and localities. One of the first published works on the island described an ethnobotanical survey conducted by Wong [6] over 40 years ago in Blanchisseuse, a small remote village, in north Trinidad. This was followed by a more extensive survey of medicinal plants undertaken between 1979 and 1980 conducted by Seaforth and colleagues across 18 localities on the island. The findings of this survey resulted in the publication of *A guide to the medicinal plants of Trinidad & Tobago* by the Commonwealth Secretariat [8]. Over the last decade, a survey by Lans used a small sample size of 30 persons across 13 sites [9]. During that period, and a group led by Clement [10–12] focused on complementary use of herbal remedies in a few hundred patients attending over 20 modern primary public healthcare facilities spread across the island, who were being managed with the chronic diseases namely, hypertension, diabetes mellitus and asthma.

Overall, the global trend indicates that knowledge of traditional folkloric medicine is fast disappearing, especially in the urban communities. Similarly, in Trinidad which is relatively industrialized most of the population reside in urban communities and have access to modern healthcare and medicines. We therefore assumed that people living in rural agricultural communities, with restricted access to transportation and healthcare facilities, would retain more traditional knowledge and more likely to use herbal remedies rather than those in urban areas. This assumption is supported by other studies [13-15], and hence the rationale for focusing our survey in these communities.

The objectives of our survey were to; i) determine the most common ailments treated with herbal remedies, ii) identify the plants used to treat these common ailments, plant part(s) used, and their mode of preparation, iii) determine which of these plants met the 20 % criteria for popular (significant) use according to TRAMIL criterion and iv) assess the literature regarding the pharmacological evidence that could support the traditional use of these popular (significant) plants.

## Materials and methods

**Fig. 1:**
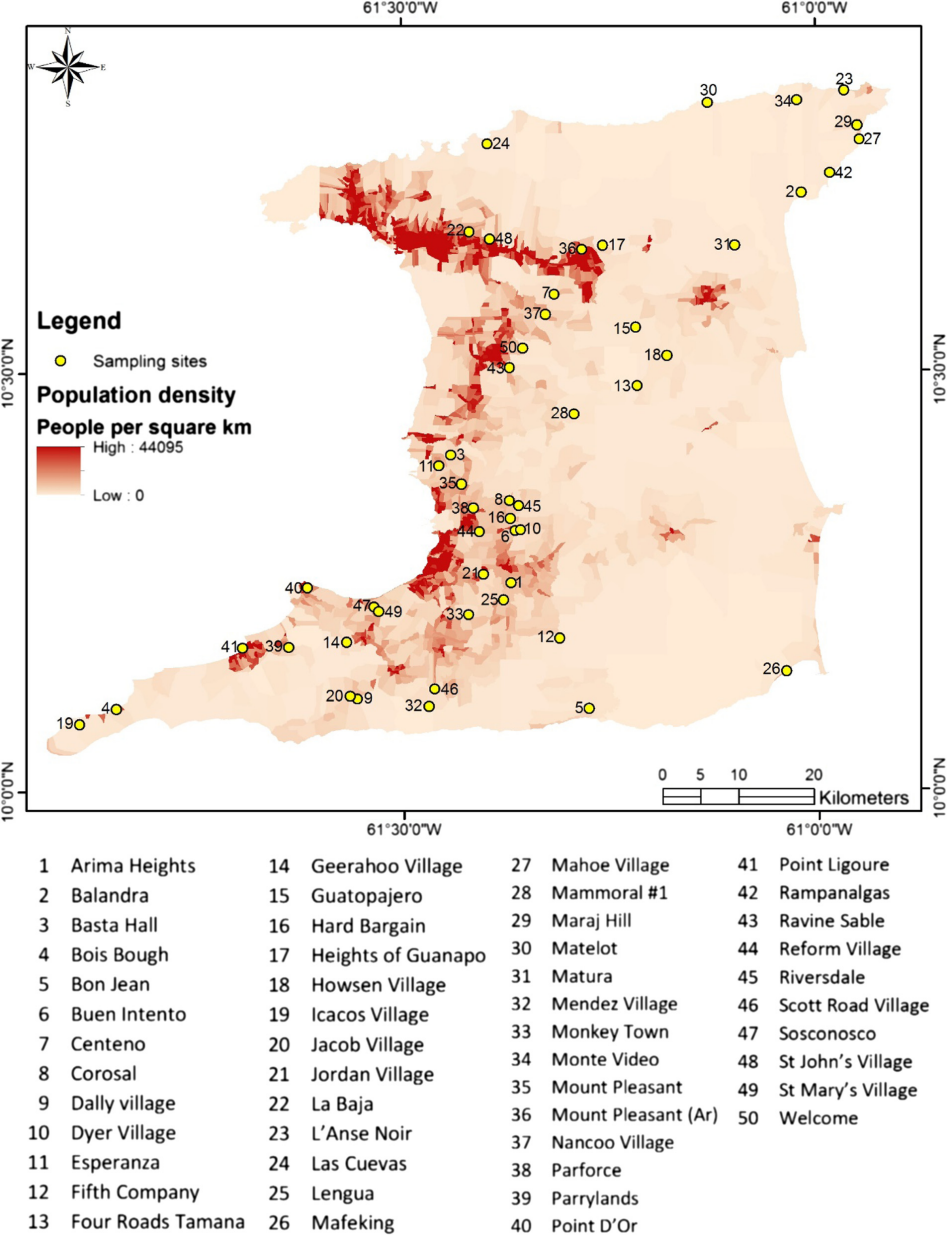
Map showing localities where ethnobotanical survey was conducted in Trinidad

An ethnobotanical survey was conducted in 450 households across 50 randomly selected rural communities on the island of Trinidad between October 2007 and July 2008 (Fig. 1). We used the TRAMIL (Traditional Medicine in the Islands) questionnaire to collect data regarding medicinal plant use Appendix 1. (See http://www.tramil.net/english/TramilModelo.html).

TRAMIL is a non-governmental network established in the Caribbean Basin since 1982 whose goals include the documentation of Caribbean folkloric knowledge of herbal medicine for the preservation of such ‘home remedy’ knowledge [16]. The network is administrated from Santo Domingo in the Dominican Republic.

### Survey instrument

The structured TRAMIL questionnaire required participants to provide information about the herbal remedies used as first treatment for the most common ailments. This instrument was used as the primary data collection tool in face-to-face interviews with respondents, following their verbal consent. The questionnaire is very simple in design and respondents were asked to describe the complaint, the plant(s) used, the preparation description, precautions and contraindications in children. The questionnaire, however, does not collect demographic details, such as sex, age, income, etc. TRAMIL defines a remedy with significant (popular) use as the combination of plant species, plant part and form of preparation that is identified by 20 % or more of the study population as a primary treatment resource for a given ailment.

### Pilot study

The pilot survey was undertaken to validate the use of the TRAMIL survey instrument in the island to determine the top ten aliments most commonly treated with ‘herbal remedies’.

Using data provided by the Central Statistical Office [1] and the Elections and Boundaries Commission [17] in Trinidad and Tobago regarding the population distribution, and the locations of healthcare facilities (provided by the Ministry of Health), we identified over 200 communities that satisfied the inclusion criteria for selection as a rural community. The criteria were

(1) having a population of less than 1500 inhabitants and (2) limited access to amenities, particularly healthcare services. The name of each community was written on equally sized pieces of paper, folded uniformly, placed in a pot and the five communities were randomly selected for the pilot study. For each community ten randomly selected households were interviewed. From each household the individual with the most knowledge about herbal medicine was interviewed. The top ten ailments most commonly treated with herbal remedies were then selected from the full range of ailments recorded from all the informants.

### Site selection and plant collection

The selection of the 50 rural communities for the wider national survey were randomly chosen using the same method as for the pilot study, and the distribution of communities across the island is given in Fig. 1. Using the detailed maps (including house location) provided by the Elections and Boundaries Commission for each randomly selected community, a house at the start of the main street was purposefully chosen to initiate the survey. At that house, field officers introduced themselves and explained the nature of the study and asked for the person (this could have been either male or female) in that household with the most knowledge about medicinal plant use. Subsequently, we used a snowball approach to obtain the sample size from each community; with this approach the preceding interviewee would identify someone else in the community whom they regarded as having had good knowledge of medicinal plant use.

The unmodified TRAMIL questionnaire was used in both the pilot and wider national survey; TRAMIL assumed that the eldest woman in the household had the most knowledge about herbal medicine use and would be the person of interest. However, the field officers asked for the person (which could be either male or female) with the most knowledge about herbal medicine. Although our field officers noted the names and ages of some of the respondents, this was not consistently collected and we therefore could not include any demographic details in our results. Respondents provided vernacular names for plants and the field interviewers collected specimens which were subsequently taken to the National Herbarium of Trinidad and Tobago for identification by a plant taxonomist. The website www.theplantlist.org was accessed to verify the accepted nomenclature for each species.

## Results

From the pilot survey the top ten ailments emerging from the informants were cough/common cold, asthma, “stoppage-of-water”, “womb infection”, kidney stones, “afterbirth”, diabetes, hypertension, “cooling/cleanser” and fever, where herbal remedies were frequently used in rural communities in Trinidad. In the wider national survey, 1590 questionnaires were completed from 450 interviews in 50 randomly selected rural communities. These included mixtures, but we present the data for only single plant remedies. Most of the mixtures contained two or more plants, and in some cases up to eight plants with additional ingredients, such as, olive oil, “soft candle” (local name for a paraffin mixture), salt and honey. A major objective of the study was to link the individual plants with relevant pharmacological evidence to support their traditional use. Hence dealing with mixtures would prove almost impossible to determine which plant was responsible for producing the biological activity to correlate with its traditional use.

**Fig. 2:**
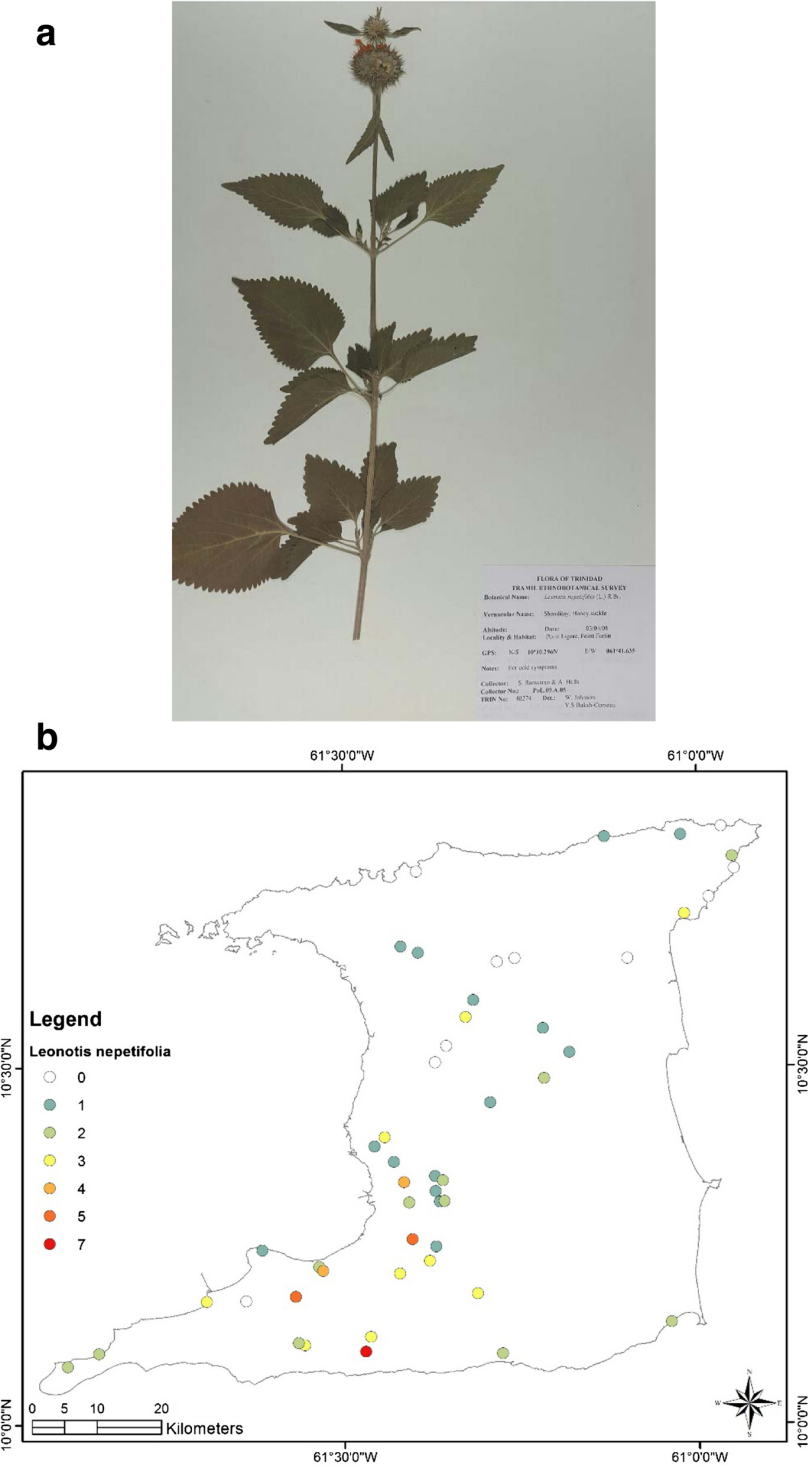
**a** (*Top*): Photograph of a voucher specimen of *Leonotis nepetifolia* (L.).R.Br. Used for cough/common cold. **b** (*Bottom*): Map of sites where *Leonotis nepetifolia* (L.) .R. Br Br. samples were collected. Used for cough/common cold

**Fig. 3:**
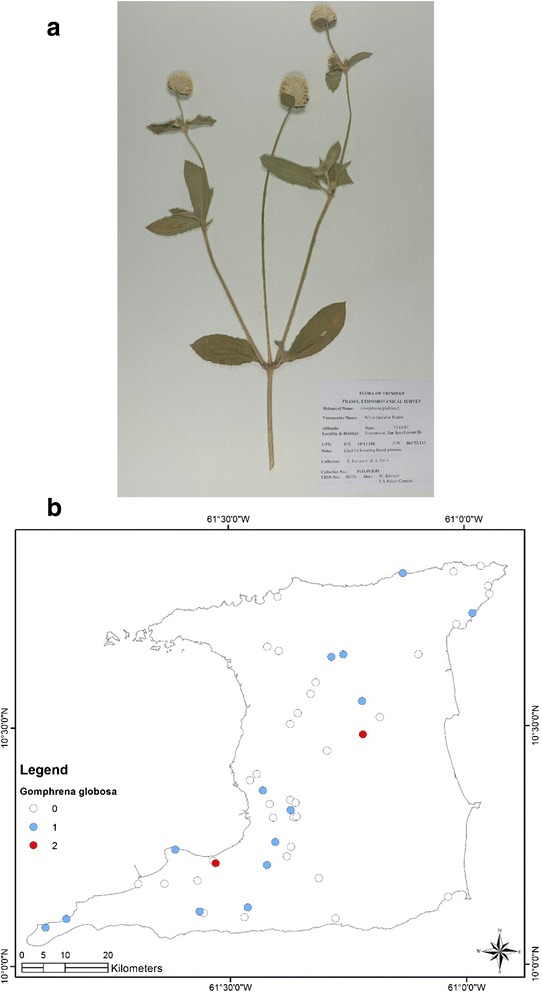
**a** (*Top*): Photograph of a voucher specimen for *Gomphrena globosa* L. Used for “stoppage of water”. **b** (*Bottom*): Map of sites where *Gomphrena globosa* L. was collected. Used for “stoppage of water”

**Fig. 4:**
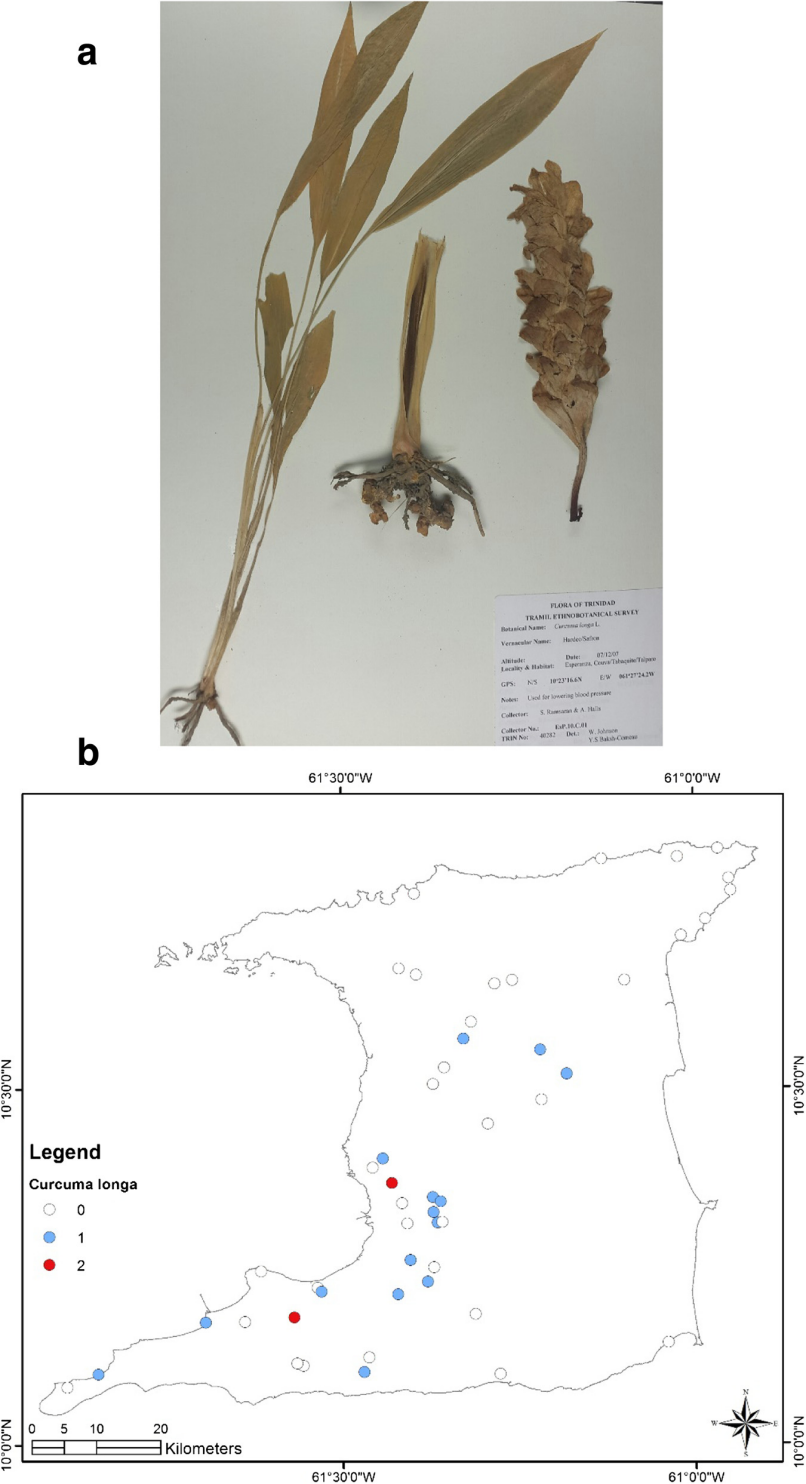
**a** (*Top*): Photograph of a voucher specimen for *Curcuma longa* L. Used for “afterbirth”. **b** (*Bottom*): Map of sites where *Curcuma longa* L. was collected. Used for “afterbirth”

**Fig. 5:**
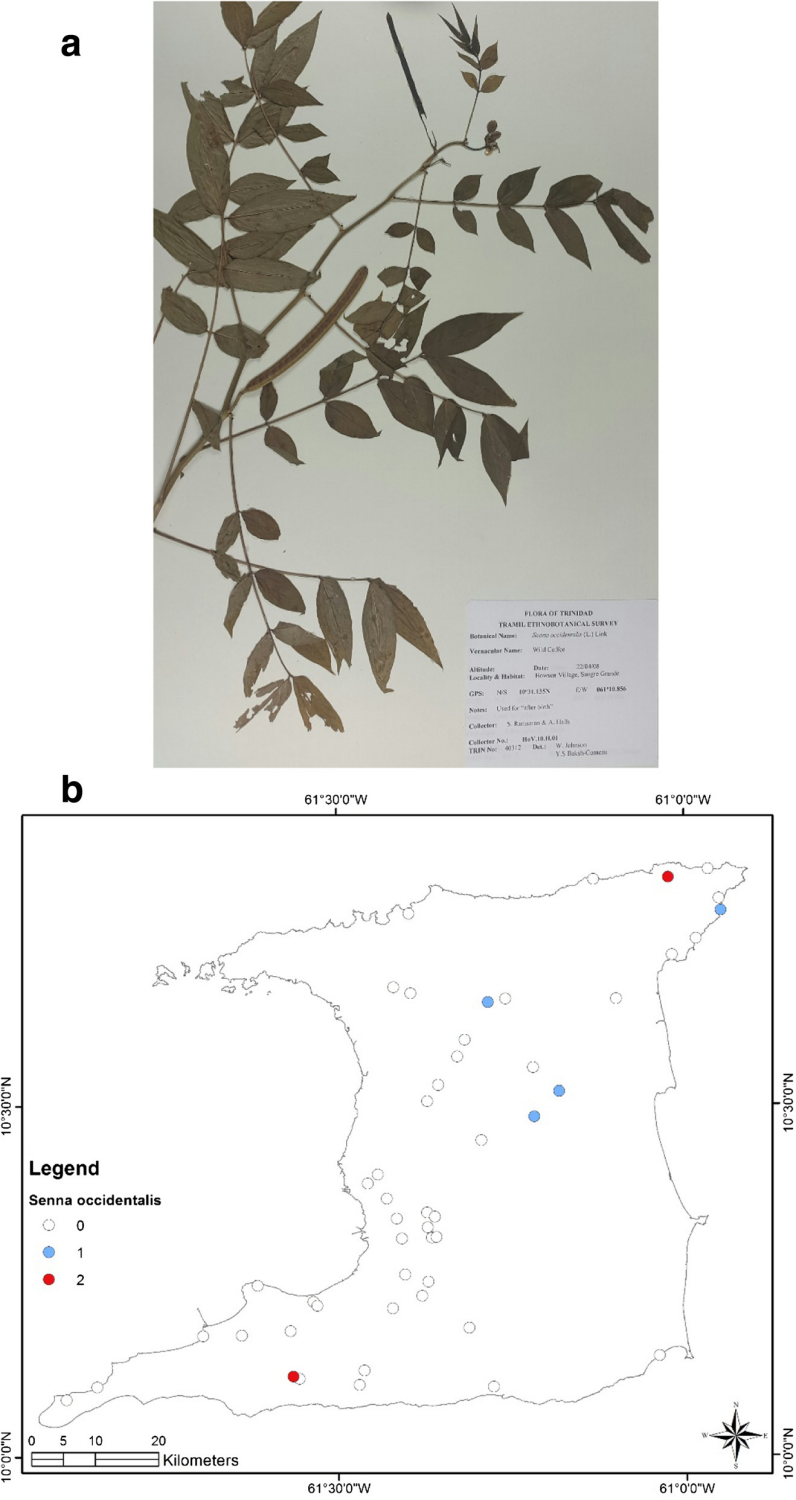
**a** (*Top*): Photograph of a voucher specimen for *Senna occidentalis* (L.) Link Link. Used for “afterbirth”. **b** (*Bottom*): Map of sites where *Senna occidentalis* (L.) Link. was collected. Used for “afterbirth”

**Fig. 6:**
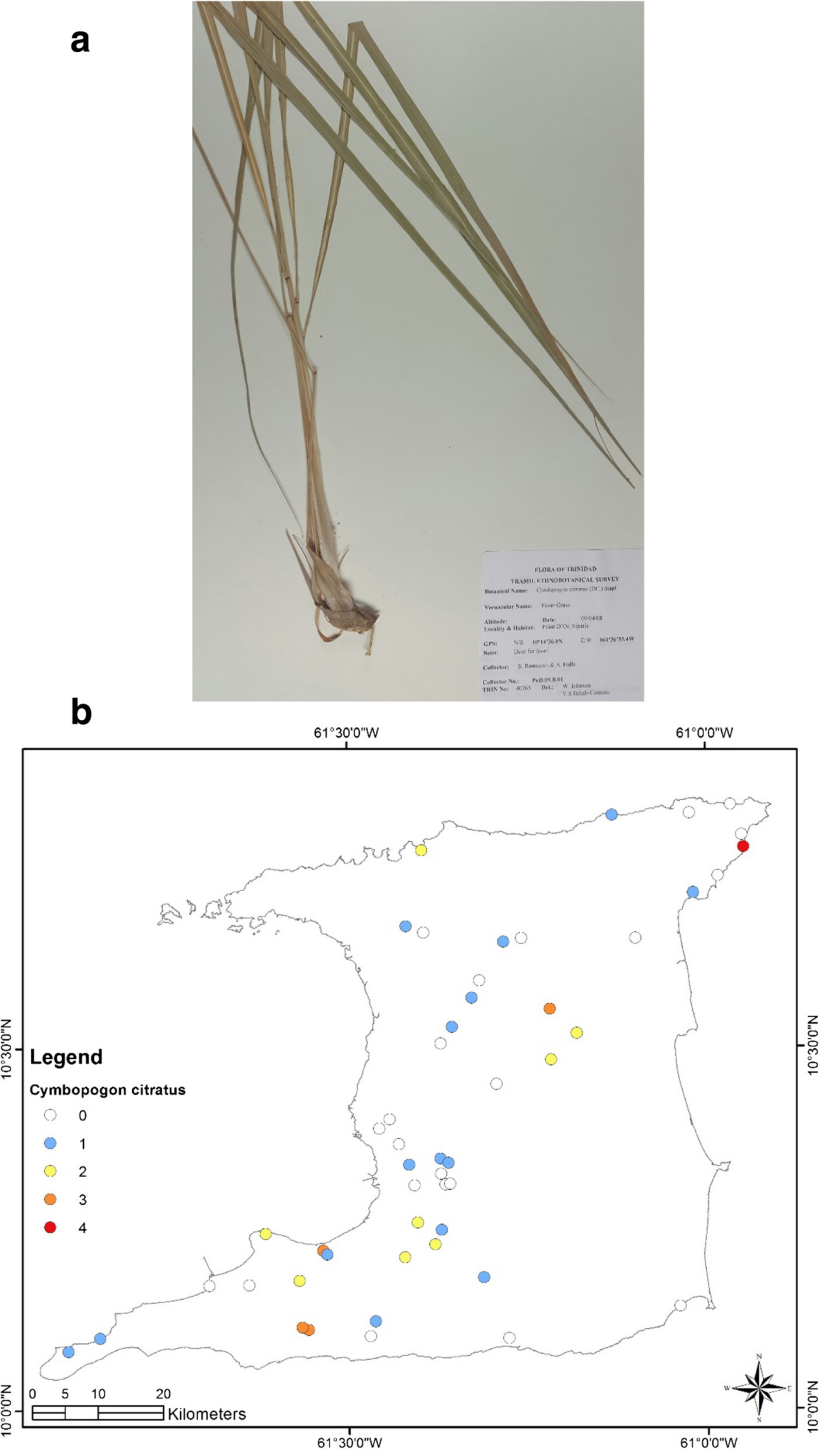
**a** (*Top*): Photograph of a voucher specimen for *Cymbopogon citratus* (DC.) Stapf Stapf. Used for fever. **b** (*Bottom*): Map of sites where *Cymbopogon citratus* (DC.) Stapf. was collected. Used for fever

**Fig. 7:**
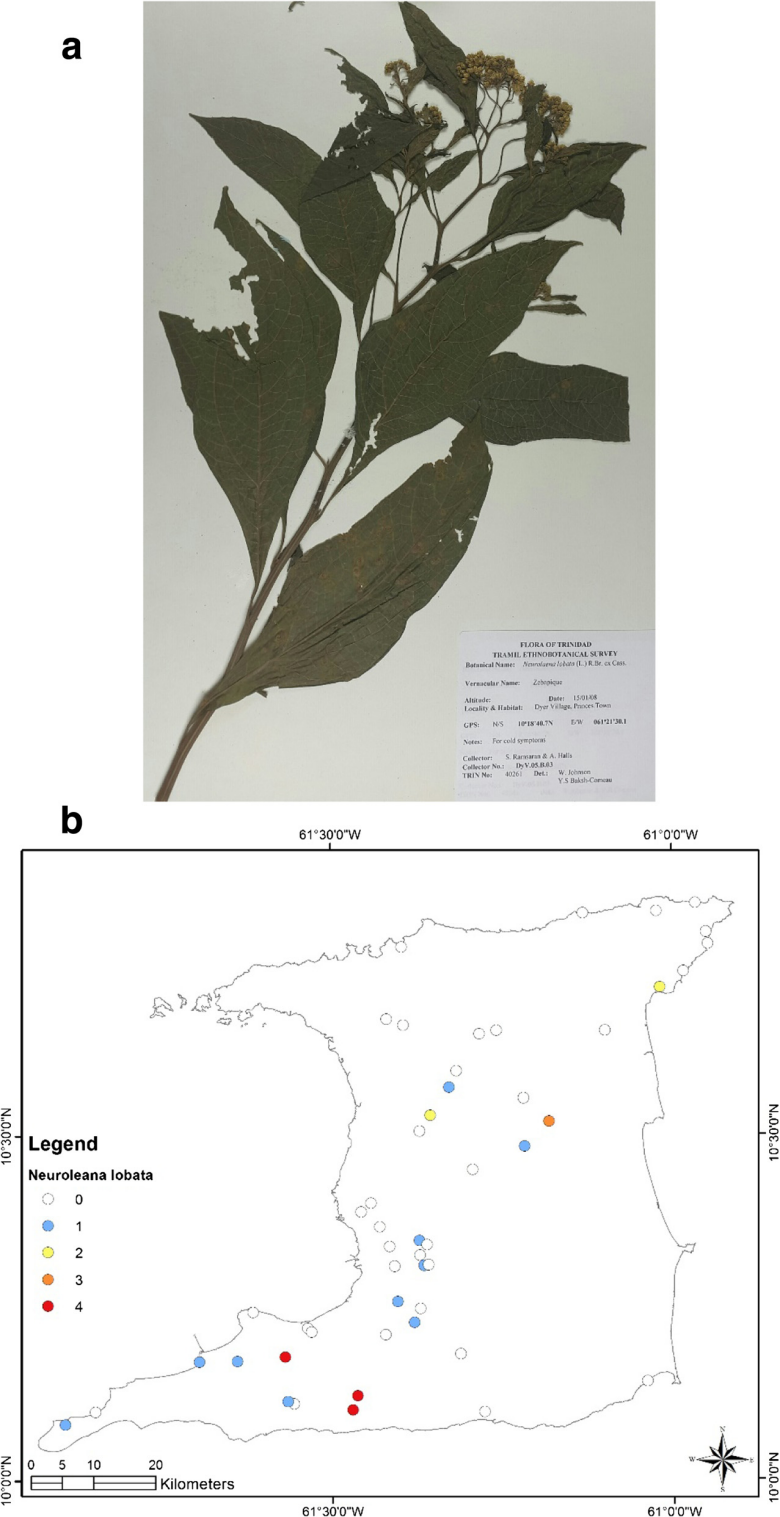
**a** (*Top*): Photograph of a voucher specimen for *Neurolaena lobata* (L.)R. Br. ex. Cass. Used for fever. **b** (*Bottom*): Map of sites where *Neurolaena lobata* (L.)R. Br. ex. Cass. was collected. Used for fever

**Fig. 8:**
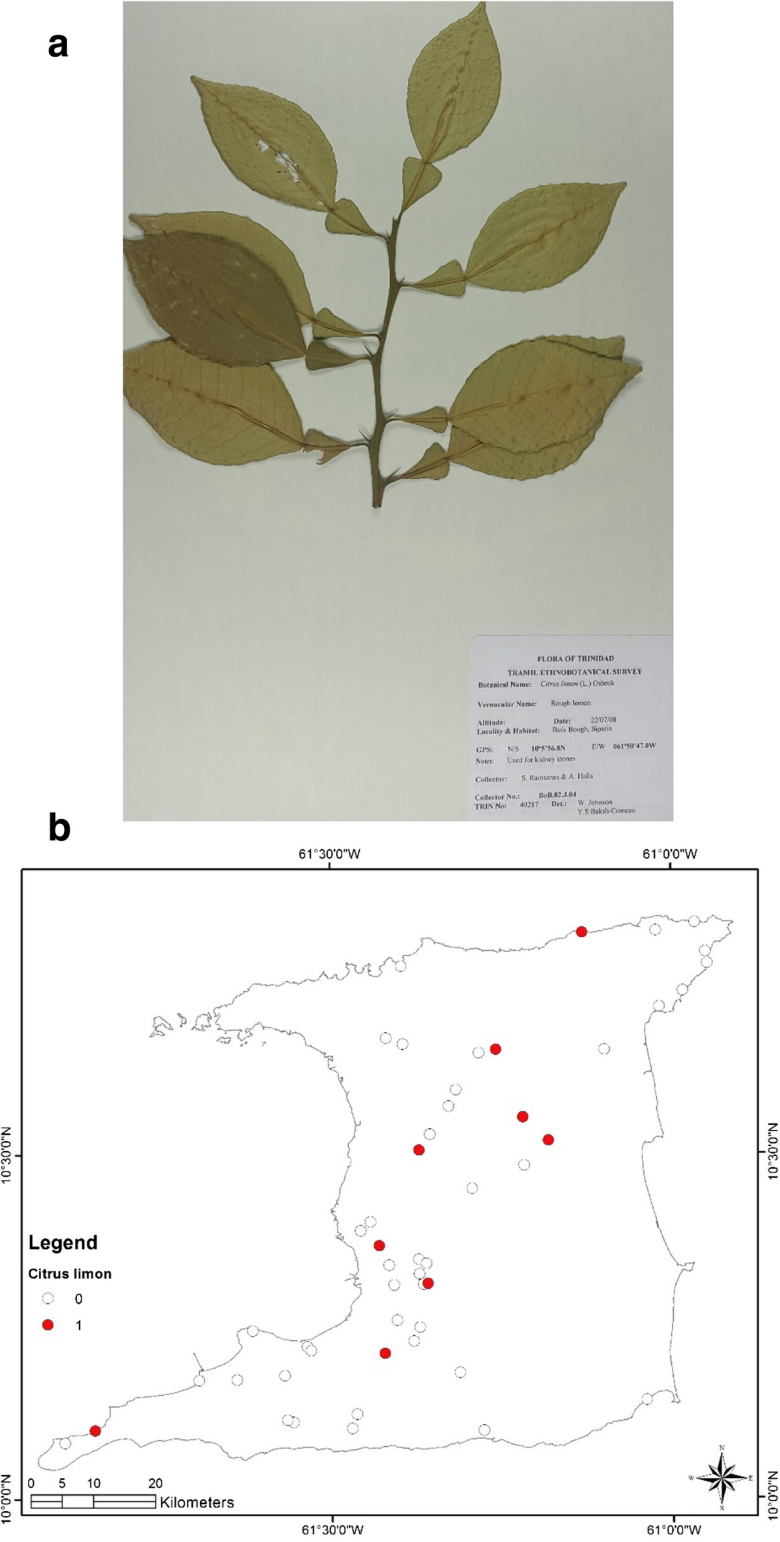
**a** (*Top*): Photograph of a voucher specimen for *Citrus limon* (L.) Osbeck. Used for kidney stones. **b** (*Bottom*): Map of sites where *Citrus limon* (L.) Osbeck. was collected. Used for kidney stones

At the end of the survey 917 single plant remedies, in different formulations, as either infusions or decoctions came from 96 species in 43 families (Table 1). The major plant families were Asteraceae (125 citations, 12 species), Lamiaceae (99 citations, 4 species), Leguminosae (88 citations, 10 species), Verbenaceae (62 citations, 3 species) and Poaceae (52 citations, 2 species). Most plant species were used for multiple ailments. The top five plant species were *Leonotis nepetifolia* (89 citations; most commonly used for cough/common cold), *Neurolaena lobata* (77 citations; most commonly used for fever), *Cymbopogon citratus* (50 citations; most commonly used for fever), *Momordica charantia* (44 citations; most commonly used for “cooling/cleanser”) and *Stachytarpheta jamaicensis* (38 citations, mostly for “cooling/cleanser’), Table 2. According to TRAMIL methodology significant plants (with 20 % or more citations by respondents for a specific ailment) were *Leonotis nepetifolia* for cough/common cold (Fig. 2a shows voucher specimen and Fig. 2b shows localities), *Gomphrena globosa* (Fig. 3a shows voucher specimen and Fig. 3b shows localities) for “stoppage of water”, *Curcuma longa* (Fig. 4a voucher specimen and Fig. 4b shows localities) and *Senna occidentalis* (Fig. 5a voucher specimen and Fig. 5b shows localities) for “afterbirth”, *Cymbopogon citratus* (Fig. 6a shows voucher specimen and Fig. 6b shows localities) and *Neurolaena lobata* (Fig. 7a shows voucher specimen and Fig. 7b shows localities) for fever, and *Citrus limon* (Fig. 8a shows voucher specimen and Fig. 8b shows localities) for kidney stones. *Stachytarpheta jamaicensis, Senna alata, Momordica charantia* and *Tournefortia hirsutissima* (although not reaching significant use by TRAMIL criterion) were also widely used as “‘cooling/cleanser’.

**Table 1:** List of plants collected in ethnobotanical survey in Trinidad

TRIN Voucher number	Family	Species	Local names	Part(s) used and preparation	Administration	Condition treated	No. of informants
40251	Acanthaceae	*Justicia pectoralis* Jacq.	Carpenter bush, St. John’s bush	Stem and leaves, decoction	Oral	Common cold & cough	14
	Acanthaceae	*Justicia pectoralis* Jacq.	Carpenter bush, St. John’s bush	Stem and leaves, decoction	Oral	Cooling/cleanser	4
40242	Acanthaceae	*Justicia secunda* Vahl	St. John’s bush	Leaves; decoction	Oral	Afterbirth	1
40241	Acanthaceae	*Thunbergia alata* Boj. ex Sims	Yellow flower vine	Flowers; infusion	Oral	Kidney stones	1
40245	Amaranthaceae	*Achyranthes indica* (L.) Mill.	Man-better-man	Leaves, soak in water and bathe	Topical	Fever	1
	Amaranthaceae	*Achyranthes indica* (L.) Mill.	Man-better-man	Leaves; decoction	Oral	Womb infection	1
40236	Amaranthaceae	*Dysphania ambrosioides* (L.) Mosyakin & Clemants	Worm grass	Stem and leaves, decoction	Oral	Fever	1
	Amaranthaceae	*Dysphania ambrosioides* (L.) Mosyakin & Clemants	Worm grass	Stem and leaves, decoction	Oral	Cooling/cleanser	1
40246	Amaranthaceae	*Gomphrena globosa* L.	White bachelor button	Flowers; decoction	Oral	Diabetes	1
	Amaranthaceae	*Gomphrena globosa* L.	White bachelor button	Flowers; infusion or decoction	Oral	Stoppage of water	17
	Amaranthaceae	*Gomphrena globosa* L.	White bachelor button	Flowers; infusion or decoction	Oral	Kidney stones	8
	Amaranthaceae	*Gomphrena globosa* L.	White bachelor button	Flowers; infusion or decoction	Oral	Womb infection	1
40243	Anacardiaceae	*Spondias mombin* L.	Hog plum	Leaves; boil and add to bath water	Topical	Cooling/cleanser	1
	Anacardiaceae	*Spondias mombin* L.	Hog plum	Leaves; as above and drink decoction after bath	Topical/Oral	Womb infection	1
	Anacardiaceae	*Spondias mombin* L.	Hog plum	Leaves; steaming decoction placed in container and mother sits over	Topical	Afterbirth	7
40244	Anacardiaceae	*Mangifera indica* L.	Mango vere	Bark; decoction	Oral	High blood pressure	1
40248	Annonaceae	*Annona muricata* L.	Soursop	Leaves, infusion or decoction	Oral	Cooling/cleanser	4
	Annonaceae	*Annona muricata* L.	Soursop	Leaves, infusion or decoction	Oral	High blood pressure	8
40248	Apocynaceae	*Catharantus roseus* (L.) G.Don	Old maid, periwinkle	Leaves and flowers; infusion or chew and swallow	Oral	Diabetes	7
	Apocynaceae	*Catharantus roseus* (L.) G.Don	Old maid, periwinkle	Leaves and flowers; infusion or chew and swallow	Oral	Stoppage of water	1
40250	Aristolochiaceae	*Aristolochia rugosa* Lam.	Matt root	Roots, decoction or soak in alcohol	Oral	Fever	1
	Aristolochiaceae	*Aristolochia rugosa* Lam.	Matt root	Roots, decoction or soak in alcohol	Oral	Common cold & cough	1
	Aristolochiaceae	*Aristolochia rugosa* Lam.	Matt root	Roots, decoction or soak in alcohol	Oral	Diabetes	1
40249	Aristolochiaceae	*Aristolochia rugosa* Lam.	Tref	Leaves, chew raw leaves or soak in alcohol with caterpillar that feeds on leaves	Oral	Fever	1
	Aristolochiaceae	*Aristolochia rugosa* Lam.	Tref	Leaves, chew raw leaves or soak in alcohol with caterpillar that feeds on leaves	Oral	Common cold & cough	3
40253	Asteraceae	*Ageratum conyzoides* L*.*	Zebafam	Leaves, stem and flowers; decoction	Oral	Common cold & cough	1
	Asteraceae	*Ageratum conyzoides* L*.*	Zebafam	Leaves, stem and flowers; decoction	Oral	Cooling/cleanser	1
	Asteraceae	*Ageratum conyzoides* L*.*	Zebafam	Leaves, stem and flowers; decoction	Oral	Womb infection	4
	Asteraceae	*Ageratum conyzoides* L*.*	Zebafam	Leaves, stem and flowers; decoction	Oral	Afterbirth	2
40255	Asteraceae	*Ambrosia peruviana* Willd.	Altamis	Leaves; infusion or steamed and woman sits over	Oral/Topical	Womb infection	2
	Asteraceae	*Ambrosia peruviana* Willd.	Altamis	Leaves; infusion	Oral	Afterbirth	1
40252	Asteraceae	*Ayapana triplinervis* (Vahl) R.M. King & H.Rob.	Japana	Leaves; infusion	Oral	Common cold & cough	1
40256	Asteraceae	*Bidens pilosa* L.	Railway daisy, rabbit grass. Needle grass	Leaves, Decoction or chew and swallow juice	Oral	Common cold & cough	1
	Asteraceae	*Bidens pilosa* L.	Railway daisy, rabbit grass. Needle grass	Leaves, Decoction or chew and swallow juice	Oral	High blood pressure	6
	Asteraceae	*Bidens pilosa* L.	Railway daisy, rabbit grass. Needle grass	Leaves, Decoction or chew and swallow juice	Oral	Diabetes	2
	Asteraceae	*Bidens pilosa* L.	Railway daisy, rabbit grass. Needle grass	Leaves, Decoction or chew and swallow juice	Oral	Stoppage of water	1
40254	Asteraceae	*Chaptalia nutans* (L.) Polák	Doblan	Whole plant; infusion	Oral	High blood pressure	1
40257	Asteraceae	*Chromolaena odorata* (L.) R.M.King & H.Rob.	Christmas bush	Leaves, infusion or decoction or crush leaves and drink juice	Oral	Common cold & cough	7
	Asteraceae	*Chromolaena odorata* (L.) R.M.King & H.Rob.	Christmas bush	Leaves, infusion or decoction or crush leaves and drink juice	Oral	High blood pressure	1
40258	Asteraceae	*Eclipta prostrata* (L.) L.	Congolalla	Stem and leaves; decoction	Oral	Cooling/cleanser	2
	Asteraceae	*Eclipta prostrata* (L.) L.	Congolalla	Stem and leaves; decoction	Oral	Diabetes	2
	Asteraceae	*Eclipta prostrata* (L.) L.	Congolalla	Stem and leaves; decoction	Oral	Asthma	1
40260	Asteraceae	*Hebeclinium macrophyllum* DC.	Zebchat	Leaves; pounded in cloth and inserted in vagina	Topical	Womb infection	1
40261	Asteraceae	*Neurolaena lobata* (L.) Cass.	Zebapique	Leaves, infusion or decoction or soak in alcohol or crush leaves and drink juice	Oral	Fever	30
	Asteraceae	*Neurolaena lobata* (L.) Cass.	Zebapique	Leaves, infusion or decoction or soak in alcohol or crush leaves and drink juice	Oral	Common cold & cough	43
	Asteraceae	*Neurolaena lobata* (L.) Cass.	Zebapique	Leaves, infusion or decoction or soak in alcohol or crush leaves and drink juice	Oral	Diabetes	2
	Asteraceae	*Neurolaena lobata* (L.) Cass.	Zebapique	Leaves, infusion or decoction or soak in alcohol or crush leaves and drink juice	Oral	Womb infection	2
40262	Asteraceae	*Parthenium hysterophorus* L*.*	White top	Leaves; decoction	Oral	Diabetes	1
40263	Asteraceae	*Pluchea carolinensis* (Jacq.) G.Don	Geritout, pressure bush	Leaves, infusion or decoction	Oral	Fever	2
	Asteraceae	*Pluchea carolinensis* (Jacq.) G.Don	Geritout, pressure bush	Leaves, infusion or decoction	Oral	Common cold & cough	5
	Asteraceae	*Pluchea carolinensis* (Jacq.) G.Don	Geritout, pressure bush	Leaves, infusion or decoction	Oral	High blood pressure	1
40264	Asteraceae	*Vernonia amygdalina* Delile	Africana	Leaves; chewed and juice swallowed	Oral	High blood pressure	1
	Asteraceae	*Vernonia amygdalina* Delile	Africana	Leaves; chewed and juice swallowed	Oral	Diabetes	1
40229	Begoniaceae	*Begonia humilis* Dryand.	Lozei	Leaves, decoction	Oral	Common cold & cough	1
	Begoniaceae	*Begonia humilis* Dryand.	Lozei	Leaves, decoction	Oral	Stoppage of water	1
40227	Bignoniaceae	*Crescentia cujete* L.	Calabash	Fruit (guts); decoction with honey to make thick “lock”	Oral	Common cold & cough	1
	Bignoniaceae	*Crescentia cujete* L.	Calabash	Fruit (pulp); decoction with honey to make thick “lock”	Oral	High blood pressure	3
	Bignoniaceae	*Crescentia cujete* L.	Calabash	Leaves; infusion	Oral	Diabetes	1
	Bignoniaceae	*Crescentia cujete* L.	Calabash	Leaves; infusion	Oral	Asthma	1
40228	Bignoniaceae	*Dolichandra unguis-cati* (L.) L.G.Lohmann	Cat’s claw	Stem and leaves; hot or cold infusion, decoction	Oral	Common cold & cough	1
	Bignoniaceae	*Dolichandra unguis-cati* (L.) L.G.Lohmann	Cat’s claw	Stem and leaves; hot or cold infusion, decoction	Oral	Cooling/cleanser	3
	Bignoniaceae	*Dolichandra unguis-cati* (L.) L.G.Lohmann	Cat’s claw	Stem and leaves; hot or cold infusion, decoction	Oral	Kidney stones	2
	Bignoniaceae	*Dolichandra unguis-cati* (L.) L.G.Lohmann	Cat’s claw	Stem and leaves; hot or cold infusion, decoction	Oral	Diabetes	1
40357	Boraginacece	*Tournefortia hirsutissima* L.	Jigger bush	Stem and leaves; hot or cold infusion, decoction	Oral	Common cold & cough	1
	Boraginacece	*Tournefortia hirsutissima* L.	Jigger bush	Stem and leaves; hot or cold infusion, decoction	Oral	Cooling/cleanser	20
	Boraginacece	*Tournefortia hirsutissima* L.	Jigger bush	Stem and leaves; hot or cold infusion, decoction	Oral	Diabetes	1
40226	Bixaceae	*Bixa orellana* L.	Roucou	Leaves, fruits and roots; decoction	Oral	High blood pressure	1
	Bixaceae	*Bixa orellana* L.	Roucou	Leaves, fruits and roots; decoction	Oral	Diabetes	4
40225	Boraginaceae	*Cordia curassavica* (Jacq.) Roem. & Schult.	Black sage, blister bush	Leaves, infusion or crush and drink juice	Oral	Fever	1
	Boraginaceae	*Cordia curassavica* (Jacq.) Roem. & Schult.	Black sage, blister bush	Leaves, infusion or crush and drink juice	Oral	Common cold & cough	7
	Boraginaceae	*Cordia curassavica* (Jacq.) Roem. & Schult.	Black sage, blister bush	Leaves, infusion or crush and drink juice	Oral	Cooling/cleanser	1
	Boraginaceae	*Cordia curassavica* (Jacq.) Roem. & Schult.	Black sage, blister bush	Leaves (yellow); decoction	Oral	Womb infection	1
40231	Cactaceae	*Nopalea cochenillifera* (L.) Salm-Dyck	Ratchet	Succulent leaves; grate, hot or cold infusion	Oral	Cooling/cleanser	9
40230	Cactaceae	*Rhipsalis baccifera* (J.S.Muell.) Stearn	Old man beard	Stem and leaves; infusion or decoction	Oral	Diabetes	3
40240	Caprifoliaceae	*Sambucus canadensis* L*.*	Serrio	Leaves and flowers, decoction or crush and drink juice (add olive oil with/without salt)	Oral	Fever	1
	Caprifoliaceae	*Sambucus canadensis* L*.*	Serrio	Leaves and flowers, decoction or crush and drink juice (add olive oil with/without salt)	Oral	Common cold & cough	20
	Caprifoliaceae	*Sambucus canadensis* L*.*	Serrio	Leaves and flowers, decoction or crush and drink juice (add olive oil with/without salt)	Oral	Asthma	2
40238 (40239)	Caricaceae	*Carica papaya* L*.*	Pawpaw	Fruit (young); grated for infusion or decoction	Oral	High blood pressure	9
	Caricaceae	*Carica papaya* L*.*	Pawpaw	Leaves; chew and swallow juice	Oral	Diabetes	2
	Caricaceae	*Carica papaya* L*.*	Pawpaw	Roots of male plant; infusion	Oral	Stoppage of water	1
	Caricaceae	*Carica papaya* L*.*	Pawpaw	Roots of male plant; decoction	Oral	Kidney stones	2
40235	Commelinaceae	*Commelina erecta* L*.*	Watergrass	Stem and leaves; infusion or decoction	Oral	Cooling/cleanser	6
	Commelinaceae	*Commelina erecta* L*.*	Watergrass	Stem and leaves; infusion or decoction	Oral	Stoppage of water	2
40233	Convolvulaceae	*Cuscuta campestris* Yunck.	Love vine	Vine (stem and leaves); infusion or decoction	Oral	Cooling/cleanser	3
	Convolvulaceae	*Cuscuta campestris* Yunck.	Love vine	Vine (stem and leaves); infusion or decoction	Oral	Kidney stones	1
40232	Crassulaceae	*Bryophyllum pinnatum* (Lam.) Oken	Wonder-of-the-world	Leaves, infusion or heat leaves and squeeze juice	Oral	Common cold & cough	8
	Crassulaceae	*Bryophyllum pinnatum* (Lam.) Oken	Wonder-of-the-world	Leaves, infusion or heat leaves and squeeze juice	Oral	Cooling/cleanser	2
	Crassulaceae	*Bryophyllum pinnatum* (Lam.) Oken	Wonder-of-the-world	Leaves, infusion or heat leaves and squeeze juice	Oral	High blood pressure	2
	Crassulaceae	*Bryophyllum pinnatum* (Lam.) Oken	Wonder-of-the-world	Leaves, infusion or heat leaves and squeeze juice	Oral	Diabetes	5
	Crassulaceae	*Bryophyllum pinnatum* (Lam.) Oken	Wonder-of-the-world	Leaves, infusion or heat leaves and squeeze juice	Oral	Asthma	2
	Crassulaceae	*Bryophyllum pinnatum* (Lam.) Oken	Wonder-of-the-world	Leaves, infusion or heat leaves and squeeze juice	Oral	Kidney stones	1
40231	Cucurbitaceae	*Momordica charantia* L.	Caraille	Vine (stem and leaves), decoction	Oral	Fever	2
	Cucurbitaceae	*Momordica charantia* L.	Caraille	Vine (stem and leaves), decoction	Oral	Common cold & cough	2
	Cucurbitaceae	*Momordica charantia* L.	Caraille	Vine (stem and leaves), decoction	Oral	Cooling/cleanser	21
	Cucurbitaceae	*Momordica charantia* L.	Caraille	Vine (stem and leaves), decoction	Oral	High blood pressure	4
	Cucurbitaceae	*Momordica charantia* L.	Caraille	Vine (stem and leaves), decoction	Oral	Diabetes	7
	Cucurbitaceae	*Momordica charantia* L.	Caraille	Vine (stem and leaves), decoction	Oral	Womb infection	7
	Cucurbitaceae	*Momordica charantia* L.	Caraille	Vine (stem and leaves), decoction	Oral	Afterbirth	1
40267	Euphorbiaceae	*Euphorbia thymifolia* L.	Female malomay	Vine (stem and leaves); infusion	Oral	High blood pressure	1
40268	Euphorbiaceae	*Euphorbia hirta* L*.*	Malomay	Leaves; infusion	Oral	Kidney stones	1
	Euphorbiaceae	*Euphorbia hirta* L*.*	Malomay	Leaves; infusion	Oral	Womb infection	1
40273	Euphorbiaceae	*Euphorbia oerstediana* (Klotzsch & Garcke) Boiss.	Kidney bush	Leaves, stem, seeds; decoction	Oral	Kidney stones	2
40272	Euphorbiaceae	*Jatropha gossypiifolia* L. var. *elegans* (Pohl) Müll.Arg	Red physic nut, wild cassava	Leaves; decoction or apply leaves to body	Topical	Common cold & cough	1
	Euphorbiaceae	*Jatropha gossypiifolia* L. var. *elegans* (Pohl) Müll.Arg	Red physic nut, wild cassava	Leaves; decoction or apply leaves to body	Oral	Cooling/cleanser	1
	Euphorbiaceae	*Jatropha gossypiifolia* L. var. *elegans* (Pohl) Müll.Arg	Red physic nut, wild cassava	Leaves; decoction or apply leaves to body	Oral	Womb infection	1
40234	Gentianaceae	*Enicostema verticillatum* (L.) Engl. ex Gilg.	Kainnine	Leaves, chew and swallow juice	Oral	Fever	1
40277	Lamiaceae	*Hyptis suaveolens* (L.) Poit.	African mint, jungle mint, matram, hyssop	Leaves, infusion or crush and inhaled	Oral, Inhalation	Fever	1
	Lamiaceae	*Hyptis suaveolens* (L.) Poit.	African mint, jungle mint, matram, hyssop	Leaves, infusion or crush and inhaled	Oral	Common cold & cough	3
	Lamiaceae	*Hyptis suaveolens* (L.) Poit.	African mint, jungle mint, matram, hyssop	Leaves, infusion or crush and inhaled	Oral	Cooling/cleanser	1
40274	Lamiaceae	*Leonotis nepetifolia* (L.) R.Br.	Shandilay	Leaves, infusion or decoction or pound and squeeze juice (add salt)	Oral	Fever	1
	Lamiaceae	*Leonotis nepetifolia* (L.) R.Br.	Shandilay	Leaves, infusion or decoction or pound and squeeze juice (add salt)	Oral	Common cold & cough	85
	Lamiaceae	*Leonotis nepetifolia* (L.) R.Br.	Shandilay	Leaves, infusion or decoction or pound and squeeze juice (add salt)	Oral	Cooling/cleanser	1
	Lamiaceae	*Leonotis nepetifolia* (L.) R.Br.	Shandilay	Leaves, infusion or decoction or pound and squeeze juice (add salt)	Oral	Diabetes	1
	Lamiaceae	*Leonotis nepetifolia* (L.) R.Br.	Shandilay	Leaves, infusion or decoction or pound and squeeze juice (add salt)	Oral	Asthma	1
40276	Lamiaceae	*Ocimum gratissimum* L.	Aroubaba	Leaves and stem, decoction	Oral	Fever	1
	Lamiaceae	*Ocimum gratissimum* L.	Aroubaba	Leaves and stem, decoction	Oral	Common cold & cough	2
40275	Lamiaceae	*Ocimum micranthum* Willd.	Jumbie basil	Stem and leaves, infusion or decoction		Cooling/cleanser	2
40278	Lauraceae	*Persea americana Mill.*	Avocado	Leaves; decoction	Oral	High blood pressure	2
40317	Leguminosae	*Abrus precatorius* L.	Jumbie bead	Vine (stem and leaves); infusion or decoction	Oral	Common cold & cough	4
40318	Leguminosae	*Brownea coccinea* Jacq. ssp. *capitella* (Jacq.) D.Velázquez & G.Agostini,	Cooper hook	Flowers; infusion	Oral	Womb infection	1
40319	Leguminosae	*Cajanus cajan* (L.) Millsp.	Pigeon peas	Leaves; decoction added to bath water	Topical	Common cold & cough	1
	Leguminosae	*Cajanus cajan* (L.) Millsp.	Pigeon peas	Leaves; decoction added to bath water	Oral	High blood pressure	1
40320	Leguminosae	*Entada polystachya* (L.) DC.	Mayok shapel	Bark and roots; infusion	Oral	Cooling/cleanser	3
	Leguminosae	*Entada polystachya* (L.) DC.	Mayok shapel	Bark and roots; infusion	Oral	Stoppage of water	1
	Leguminosae	*Entada polystachya* (L.) DC.	Mayok shapel	Bark and roots; infusion	Oral	Womb infection	1
40315	Leguminosae	*Flemingia strobilifera* (L.) W.T.Aiton	Mosquito bush, wild hops	Whole plant (with roots); decoction	Oral	Stoppage of water	2
	Leguminosae	*Flemingia strobilifera* (L.) W.T.Aiton	Mosquito bush, wild hops	Whole plant (with roots); decoction	Oral	Kidney stones	3
40316	Leguminosae	*Mimosa pudica* L.	Timarie, shame bush, sensitive plant, Mary-Mary- close- your- door	Whole plant; decoction	Oral	Common cold & cough	1
	Leguminosae	*Mimosa pudica* L.	Timarie, shame bush, sensitive plant, Mary-Mary- close- your- door	Whole plant; decoction	Oral	Diabetes	1
	Leguminosae	*Mimosa pudica* L.	Timarie, shame bush, sensitive plant, Mary-Mary- close- your- door	Roots; decoction	Oral	Stoppage of water	4
	Leguminosae	*Mimosa pudica* L.	Timarie, shame bush, sensitive plant, Mary-Mary- close- your- door	Roots; decoction	Oral	Kidney stones	6
	Leguminosae	*Mimosa pudica* L.	Timarie, shame bush, sensitive plant, Mary-Mary- close- your- door	Roots; decoction	Oral	Womb infection	3
40304 (36911)	Leguminosae	*Senna alata* (L.) Roxb.	Wild senna, ringworm bush	Leaves; infusion or decoction	Oral	Cooling/cleanser	26
	Leguminosae	*Senna alata* (L.) Roxb.	Wild senna, ringworm bush	Seeds; roasted, grounded and infusion	Oral	Asthma	1
	Leguminosae	*Senna alata* (L.) Roxb.	Wild senna, ringworm bush	Leaves and flowers; infusion or decoction	Oral	Womb infection	3
40314	Leguminosae	*Senna bacillaris* (L.f.) H.S.Irwin & Barneby	Christmas bush	Leaves and stem; decoction	Oral	Kidney stones	1
40312	Leguminosae	*Senna occidentalis* (L.) Link	Wild coffee	Seeds; roasted, grounded and infusion	Oral	Common cold & cough	1
	Leguminosae	*Senna occidentalis* (L.) Link	Wild coffee	Roots; decoction	Oral	Cooling/cleanser	1
	Leguminosae	*Senna occidentalis* (L.) Link	Wild coffee	Roots; decoction	Oral	Asthma	1
	Leguminosae	*Senna occidentalis* (L.) Link	Wild coffee	Roots; decoction	Oral	Womb infection	1
	Leguminosae	*Senna occidentalis* (L.) Link	Wild coffee	Roots; decoction	Oral	Afterbirth	8
40313	Leguminosae	*Tamarindus indica* L.	Tambran	Fruit (with seeds) and leaves; infusion	Oral	High blood pressure	11
	Leguminosae	*Tamarindus indica* L.	Tambran	Bark; decoction	Oral	Diabetes	1
	Leguminosae	*Tamarindus indica* L.	Tambran	Bark; decoction	Oral	Asthma	1
40280	Loranthaceae	*Phthirusa stelis* (L.) Kuijt	Birdvine	Vine (stem and leaves); decoction	Oral	Stoppage of water	1
40310	Malvaceae	*Hibiscus rosa-sinensis* L.	Double hibiscus, Arrahoo	Flowers, infusion or decoction	Oral	Common cold & cough	10
	Malvaceae	*Hibiscus rosa-sinensis* L.	Double hibiscus, Arrahoo	Flowers, infusion or decoction	Oral	Cooling/cleanser	1
	Malvaceae	*Hibiscus rosa-sinensis* L.	Double hibiscus, Arrahoo	Flowers, infusion or decoction	Oral	Diabetes	1
	Malvaceae	*Hibiscus rosa-sinensis* L.	Double hibiscus, Arrahoo	Flowers, infusion or decoction	Oral	Stoppage of water	1
	Malvaceae	*Hibiscus rosa-sinensis* L.	Double hibiscus, Arrahoo	Flowers, infusion or decoction	Oral	Womb infection	1
40311	Malvaceae	*Urena sinuata* L.	Kuzen mahoe	Stem and leaves; infusion or decoction	Oral	Cooling/cleanser	2
	Malvaceae	*Urena sinuata* L.	Kuzen mahoe	Stem and leaves; infusion or decoction	Oral	Kidney stones	1
40309	Meliaceae	*Azadirachta indica* A.Juss.	Neem	Leaves; decoction	Oral	Cooling/cleanser	1
	Meliaceae	*Azadirachta indica* A.Juss.	Neem	Leaves; chew and swallow juice	Oral	Diabetes	7
40308	Meliaceae	*Carapa guianensis* Aubl.	Carapa, crappo	Seed (oil extracted)	Oral	Common cold & cough	1
	Meliaceae	*Carapa guianensis* Aubl.	Carapa, crappo	Seed (oil extracted)	Oral	Asthma	1
40306	Menispermaceae	*Cissampelos pareira* L.	Grave yard bush, Cat ears, patacon	Vine (stem and leaves); infusion or decoction	Oral	Common cold & cough	3
	Menispermaceae	*Cissampelos pareira* L.	Grave yard bush, Cat ears, patacon	Vine (stem and leaves); infusion or decoction	Oral	Asthma	1
40307	Moraceae	*Artocarpus altilis* (Parkinson) Fosberg	Breadfruit	Yellow leaves; infusion or decoction	Oral	High blood pressure	11
40303	Moraceae	*Morus alba* L.	Pressure bush	Leaves; infusion or decoction	Oral	High blood pressure	8
40302	Myoporaceae	*Bontia daphnoides* L.	Olive bush	Leaves; infusion or decoction	Oral	Common cold & cough	1
	Myoporaceae	*Bontia daphnoides* L.	Olive bush	Leaves; infusion or decoction	Oral	Cooling/cleanser	6
	Myoporaceae	*Bontia daphnoides* L.	Olive bush	Leaves; infusion or decoction	Oral	High blood pressure	2
	Myoporaceae	*Bontia daphnoides* L.	Olive bush	Leaves; infusion or decoction	Oral	Diabetes	2
	Myoporaceae	*Bontia daphnoides* L.	Olive bush	Leaves; infusion or decoction	Oral	Kidney stones	5
	Myoporaceae	*Bontia daphnoides* L.	Olive bush	Leaves; infusion or decoction	Oral	Womb infection	1
40301	Myrtaceae	*Pimenta racemosa* (Mill.) J.W.Moore	Bay leaf, bay rum	Leaves, decoction	Oral	Fever	1
	Myrtaceae	*Pimenta racemosa* (Mill.) J.W.Moore	Bay leaf, bay rum	Leaves, decoction	Oral	Common cold & cough	1
	Myrtaceae	*Pimenta racemosa* (Mill.) J.W.Moore	Bay leaf, bay rum	Leaves, decoction	Oral	Cooling/cleanser	3
	Myrtaceae	*Pimenta racemosa* (Mill.) J.W.Moore	Bay leaf, bay rum	Leaves, decoction	Oral	High blood pressure	1
40269	Phyllanthaceae	*Phyllanthus amarus* Schumach. & Thonn.	Seed-under-leaf, Guen amber faye	Whole plant(stem, leaves and roots)	Oral	Diabetes	6
40271	Phyllanthaceae	*Phyllanthus urinaria* L.	Seed-under-leaf	Whole plant (stem, leaves and roots); decoction	Oral	Womb infection	2
	Phyllanthaceae	*Phyllanthus urinaria* L.	Seed-under-leaf	Whole plant (stem, leaves and roots); decoction	Oral	Stoppage of water	1
	Phyllanthaceae	*Phyllanthus urinaria* L.	Seed-under-leaf	Whole plant (stem, leaves and roots); decoction	Oral	High blood pressure	2
40270	Phyllanthaceae	*Phyllantus sp.*	Seed-under-leaf	Whole plant (stem, leaves and roots): infusion or decoction	Oral	Cooling	1
	Phyllanthaceae	*Phyllantus sp.*	Seed-under-leaf	Whole plant (stem, leaves and roots): infusion or decoction	Oral	Stoppage of water	1
	Phyllanthaceae	*Phyllantus sp.*	Seed-under-leaf	Whole plant (stem, leaves and roots): infusion or decoction	Oral	Kidney stones	3
40298	Phytolaccaceae	*Microtea debilis* Sw.	Alantoki	Leaves; decoction	Oral	Common cold & cough	3
	Phytolaccaceae	*Microtea debilis* Sw.	Alantoki	Whole plant (leaves, stem and roots)	Oral	Asthma	1
40297	Phytolaccaceae	*Petiveria alliacea* L.	Gully root	Roots; crushed and infused or soak in alcohol	Oral	Common cold & cough	1
40300	Passifloraceae	*Passiflora edulis* Sims	Passion fruit	Leaves; decoction	Oral	High blood pressure	1
40299	Passifloraceae	*Passiflora quadrangularis* L.	Barbadine	Leaves; infusion or decoction	Oral	High blood pressure	9
40296	Piperaceae	*Peperomia pellucida* (L.) Kunth	Shining bush	Whole plant; infusion or decoction	Oral	Common cold & cough	2
	Piperaceae	*Peperomia pellucida* (L.) Kunth	Shining bush	Whole plant; infusion or decoction	Oral	Cooling/cleanser	15
	Piperaceae	*Peperomia pellucida* (L.) Kunth	Shining bush	Whole plant; infusion or decoction	Oral	Stoppage of water	1
40295	Piperaceae	*Piper marginatum* Jacq.	Lanie bois	Leaves; decoction	Oral	Womb infection	1
40292	Piperaceae	*Piper tuberculatum* Jacq.	Candle bush	Leaves; decoction	Oral	Diabetes	1
40293	Plantaginaceae	*Plantago major* L.	Plante	Leaves; infusion	Oral	High blood pressure	1
40265	Poaceae (Gramineae)	*Cymbopogon citratus* (DC.) Stapf	Fevergrass	Leaves and roots, decoction	Oral	Fever	46
	Poaceae (Gramineae)	*Cymbopogon citratus* (DC.) Stapf	Fevergrass	Leaves and roots, decoction	Oral	Common cold & cough	1
	Poaceae (Gramineae)	*Cymbopogon citratus* (DC.) Stapf	Fevergrass	Leaves and roots, decoction	Oral	Cooling/cleanser	3
40266	Poaceae (Gramineae)	*Pennisetum purpureum* Schumach.	Wild cane, cane riviere	Stem; crush in cold infusion	Oral	Cooling/cleanser	1
	Poaceae (Gramineae)	*Pennisetum purpureum* Schumach.	Wild cane, cane riviere	Stem; crush in cold infusion	Oral	Stoppage of water	1
40294	Polygonaceae	*Antigonon leptopus* Hooker & Arn.	Coralita	Vine (stem and leaves)	Oral	Diabetes	1
40288	Rubiaceae	*Genipa americana* L.	Monkey apple	Fruit; decoction	Oral	Diabetes	1
40291	Rubiaceae	*Morinda citrifolia* L.	Noni, pain bush	Fruit; juice of ripened fruit	Oral	Cooling/cleanser	1
	Rubiaceae	*Morinda citrifolia* L.	Noni, pain bush	Fruit; juice of ripened fruit	Oral	Diabetes	1
40289	Rubiaceae	*Spermacoce verticillata* L.	White top, Fowl foot grass	Leaves; pound and boil	Oral	High blood pressure	1
	Rubiaceae	*Spermacoce verticillata* L.	White top, Fowl foot grass	Leaves; pound and boil	Oral	Diabetes	1
40290	Rutaceae	*Citrus x aurantifolia* (Christm. & Panzer) Swingle	Lime	Leaves (young), decoction	Oral	Fever	1
	Rutaceae	*Citrus x aurantifolia* (Christm. & Panzer) Swingle		Juice of fruit	Oral	Common cold & cough	4
	Rutaceae	*Citrus x aurantifolia* (Christm. & Panzer) Swingle		Leaves (young), decoction	Oral	Cooling/cleanser	1
40287	Rutaceae	*Citrus x limon* (L.) Osbeck,	Rough lemon	Juice of fruit, mixture with alcohol or coconut oil	Oral	Fever	1
	Rutaceae	*Citrus x limon* (L.) Osbeck	Rough lemon	Juice of fruit, mixture with alcohol or coconut oil	Oral	Common cold & cough	1
	Rutaceae	*Citrus x limon* (L.) Osbeck	Rough lemon	Juice of fruit, mixture with alcohol or coconut oil	Oral	Cooling/cleanser	4
	Rutaceae	*Citrus x limon* (L.) Osbeck	Rough lemon	Juice of fruit, mixture with alcohol or coconut oil	Oral	High blood pressure	1
	Rutaceae	*Citrus x limon* (L.) Osbeck	Rough lemon	Juice or skin (grated with molasses)	Oral	Stoppage of water	2
	Rutaceae	*Citrus x limon* (L.) Osbeck	Rough lemon	Juice of fruit (with olive oil)	Oral	Kidney stones	9
	Rutaceae	*Citrus x limon* (L.) Osbeck	Rough lemon	Juice of fruit (with olive oil)	Oral	Womb infection	1
40237	Urticaceae	*Cecropia peltata* L.	Bois cano	Dried leaves, infusion or decoction	Oral	Fever	1
	Urticaceae	*Cecropia peltata* L.	Bois cano	Dried leaves, infusion or decoction	Oral	Common cold & cough	9
	Urticaceae	*Cecropia peltata* L.	Bois cano	Dried leaves, infusion or decoction	Oral	High blood pressure	8
	Urticaceae	*Cecropia peltata* L.	Bois cano	Dried leaves, infusion or decoction	Oral	Diabetes	1
	Urticaceae	*Cecropia peltata* L.	Bois cano	Dried leaves, infusion or decoction	Oral	Stoppage of water	1
40285	Verbenaceae	*Lantana camara* L.	Kayakeet	Leaves, infusion or decoction	Oral	Fever	1
	Verbenaceae	*Lantana camara* L.	Kayakeet	Leaves, infusion or decoction	Oral	Common cold & cough	14
	Verbenaceae	*Lantana camara* L.	Kayakeet	Leaves, infusion or decoction	Oral	Cooling/cleanser	1
40284	Verbenaceae	*Lippia alba* (Mill.) N.E.Br.	Santa Maria	Leaves, decoction	Oral	Fever	2
	Verbenaceae	*Lippia alba* (Mill.) N.E.Br.	Santa Maria	Leaves, decoction	Oral	Common cold & cough	5
	Verbenaceae	*Lippia alba* (Mill.) N.E.Br.	Santa Maria	Leaves, decoction	Oral	Diabetes	1
40305	Verbenaceae	*Stachytarpheta jamaicensis* (L.) Vahl	Vervine	Leaves, infusion or decoction	Oral	Common cold & cough	1
	Verbenaceae	*Stachytarpheta jamaicensis* (L.) Vahl	Vervine	Leaves, infusion or decoction	Oral	Cooling/cleanser	36
	Verbenaceae	*Stachytarpheta jamaicensis* (L.) Vahl	Vervine	Leaves, infusion or decoction	Oral	High blood pressure	1
40286	Vitaceae	*Cissus verticillata* (L.) Vahl	Snake bush	Leaves, decoction	Oral	Common cold & cough	1
40279	Xanthorrhoeaceae	*Aloe vera* (L.) Burm.f.	Aloes	Leaves, extract gel and eat	Oral	Fever	1
	Xanthorrhoeaceae	*Aloe vera* (L.) Burm.f.	Aloes	Leaves, extract gel and eat	Oral	Common cold & cough	1
	Xanthorrhoeaceae	*Aloe vera* (L.) Burm.f.	Aloes	Leaves, extract gel and eat	Oral	Cooling/cleanser	8
	Xanthorrhoeaceae	*Aloe vera* (L.) Burm.f.	Aloes	Leaves, extract gel and eat	Oral	Womb infection	2
	Xanthorrhoeaceae	*Aloe vera* (L.) Burm.f.	Aloes	Leaves, extract gel and eat	Oral	Afterbirth	1
40281	Zingiberaceae	*Cheilocostus speciosus* (J.Koenig) C.D.Specht	Cane Riviere	Stem; Cut in pieces and boiled	Oral	Cooling/cleanser	1
	Zingiberaceae	*Cheilocostus speciosus* (J.Koenig) C.D.Specht	Cane Riviere	Stem; Cut in pieces and boiled	Oral	Diabetes	1
40282	Zingiberaceae	*Curcuma longa* L.	Saffron, hardi	Stem; pounded and make decoction	Oral	Cooling/cleanser	1
	Zingiberaceae	*Curcuma longa* L.	Saffron, hardi	Stem; pounded and make decoction	Oral	Womb infection	3
	Zingiberaceae	*Curcuma longa* L.	Saffron, hardi	Stem; pounded and make decoction	Oral	Afterbirth	17
40283	Zingiberaceae	*Zingiber officinale* Roscoe	Ginger	Rhizomes, crushed and decoction made	Oral	Common cold & cough	2

**Table 2:** Top five most commonly cited plants

Family	Species	Local names	Condition treated	No. of citations
Lamiaceae	*Leonotis nepetifolia (L.) R.Br.*	Shandilay	Fever	1
			Cough & common cold	85
			“cooling/cleanser”	1
			Diabetes	1
			Asthma	1
Asteraceae	*Neurolaena lobata (L.) R.Br. ex Cass.*	Zebapique	Fever	30
			Cough & common cold	43
			Diabetes	2
			“Womb infection”	2
Poaceae	*Cymbopogon citratus (DC.) Stapf*	Lemon grass	Fever	46
			Cough & common cold	1
			“cooling/cleanser”	3
Verbenaceae	*Stachytarpheta jamaicensis (L.) Vahl*	Vervine	Cough & common cold	1
			“cooling/cleanser”	36
			High blood pressure	1
Cucurbitaceae	*Momordica charantia L.*	Caraille	Fever	2
			Cough & common cold	2
			“cooling/cleanser”	21
			High blood pressure	4
			Diabetes	7
			“Womb infection”	7
			“afterbirth”	1

### Herbal remedies for ailments affecting the upper respiratory tract

A total of 279 persons identified herbal remedies for the treatment and management of cough/common cold. While 37 different plant species were identified, *Leonotis nepetifolia* turned out to be the most common with 85 citations out of 279 or 30.5 % thus applying the TRAMIL criteria it is a plant with popular use for the treatment of cough/common cold. The remedy was prepared as an aqueous infusion or decoction using fresh leaves, or the juice extracted by pounding the leaves and a teaspoon full mixed with a pinch of salt and taken orally. It was recommended that the remedy be taken for up to three days until symptomatic relief.

Only 13 respondents indicated the use of herbal remedies prepared from 11 different plant species in the treatment of asthmatic symptoms. However, the most commonly used plants were *Sambucus canadensis* and *Bryophyllum pinnatum* (2 each out of 13 citations; 15.4 %), which did not reach popular use level according to TRAMIL.

### Herbal remedies for ailments affecting the genitourinary tract

Four common ailments that affected the genitourinary tract were “stoppage of water”, “womb infection”, kidney stones and “afterbirth”. “Stoppage-of-water” refers to the condition commonly known as urinary retention. This condition may arise from neurological complications or due to obstructive causes, the most common being benign prostatic hypertrophy in men and uterine fibroids in women. In our survey, 39 persons indicated the use of 17 different plant species for the treatment of “stoppage-of-water”. The most common plant used was *Gomphrena globosa* (17 out of 39 citations, 43.6 %) and had popular use according to TRAMIL. The remedy was made using a few flowers of the plants to make an aqueous infusion or decoction.

Sexually transmitted infections, such as *Chlamydia* and gonorrhea, are the most common causes of uterine or “womb” infection. Uterine infections are also more likely to occur following childbirth. In our survey, 43 persons cited the use of herbal remedies for “womb infection” with plants from 24 species; *Momordica charantia* was the most commonly cited plant with seven persons indicating its use (or 16.3 % of respondents). The remedy is made using a handful of leaves, attached to the vine, to make a decoction which is consumed for up to three days until the infection clears. However, none of the plants cited for use in the treatment of “womb infection” reached popular use status according to TRAMIL.

A total of 46 persons indicated the use of herbal remedies from 15 different plant species for the treatment of kidney stones. The juice of the fruit of *Citrus limon* was cited by 9 persons (or 19.6 %) for the treatment of kidney stones and reached popular use according to TRAMIL.

The natural expulsion of the placenta following childbirth is referred to as the “afterbirth”. However, there is a prevailing cultural belief in Trinidad that in some women the whole “afterbirth” may not be fully expelled; a similar belief held by many cultures throughout the developing world [18–21]. Therefore, herbal remedies are used to ensure the complete expulsion of the placenta and blood clots in the few days following childbirth. Postnatal vaginal bleeding and discharge containing blood clots and uterine tissue (also called lochia) occurs naturally for a few weeks following childbirth, and this may have been traditionally interpreted to mean that the whole placenta was not delivered following childbirth.

In our survey, 38 respondents cited the use of herbal remedies made from eight different plant species for the treatment of “afterbirth”. These remedies were usually taken within one week of childbirth. The most commonly used plants were *Curcuma longa* (17 out of 38 citations, or 44.7 %) and *Senna occidentalis* (8 out of 38 citations, 21.1 %) and both reached popular use status according to TRAMIL criterion. For *Curcuma longa* the herbal remedy was prepared using the rhizome or underground stem of the plant to make either a decoction or by pounding and extracting the juice for oral consumption. The roots of *Senna occidentalis* are used to make a decoction for oral administration.

### Herbal remedies for chronic diseases

Diabetes mellitus and hypertension were the two common chronic diseases identified in the survey. For diabetes, there were 67 citations with plants being derived from 30 different species; *Catharanthus roseus*, *Momordica charantia* and *Azadirachta indica* were the most commonly cited plants for the treatment of diabetes, and were cited equally by 7 persons (10.5 % each). However, none of these plants reached significant use by TRAMIL standards.

There were 100 citations for hypertension treatment from 28 different plant species. The most commonly used plants for the treatment of hypertension were *Artocarpus altilis* (11 citations, or 11.0 %) and *Tamarindus indica* (11 citations, or 11.0 %). The yellow leaves of *Artocarpus altilis* were used to make either an infusion or decoction and an infusion or decoction was made using the fruit and leaves of *Tamarindus indica.* The remedies for diabetes and hypertension were used irregularly for symptomatic control of high blood pressure.

### Herbal remedies used for ‘cooling/cleanser” and treatment of fever

The humoral medicine concept, with the “hot-cold” dichotomy, is probably one of the oldest universally held traditional beliefs of health and disease, which has been infused into traditional cultures in Latin America and the Caribbean [22, 23]. This classical humoral concept was used to describe good health as the maintenance of equilibrium between ‘hot’ and ‘cold’ elements in the body. A disruption in this balance would result in excessively ‘hot’ or excessively ‘cold’ diseases [24]. Diseases ascribed to excessive heat were treated with ‘cold’ remedies while “cold” diseases were treated with ‘hot’ remedies [25]. It was suggested by Lans [9] that in the Trinidadian context of “hot-cold” system, traditional herbal preparations were administered in accordance with the correlation between cause and effect, with “cooling/cleanser”being used as both treatment for “hot” conditions, as well as prophylaxis to bring the body "back into balance".

Although the “hot-cold” concept of disease has long been abandoned by mainstream Western biomedical science, it remains relevant in the folkloric concept of health and disease throughout the Americas. In our survey we found a large percentage of respondents indicated the use of herbal remedies for “cooling”. Most likely the use of traditional herbal remedies as “cooling/cleanser to treat certain “hot” ailments persists in present-day Trinidad as a relic of this historical “hot-cold” dichotomy of disease. In Trinidad, as in the Americas, “hot” aliments refer to conditions such as fever, constipation, rash and skin ailments, and general malaise.

We found that 38 different plant species were used as “cooling/cleanser” by 194 persons in the survey. For most of the remedies a few fresh leaves were used to make either an infusion or decoction which was consumed for a few days up to one week. Five plants accounted for a significant 59 % of the citations: *Stachytarpheta jamaicensis* (36 citations or 18.0 %), *Senna alata* (26 citations or 13.0 %), *Momordica charantia* (21 citations or 10.5 %), *Tournefortia hirsutissima* (20 citations or 10.0 %) and *Peperomia pellucida* (15 citations or 7.5 %). However, none of these plants reached popular use status according to TRAMIL requirement.

There were 98 citations for the treatment of fever with 21 plant species being identified. Two plants, *Cymbopogon citratus* (30 out of 98 citations, 30.6 %) and *Neurolaena lobata* (46 out of 98 citations, 46.9 %) accounted for most of the citations. Most of the herbal remedies for fever were made using a few leaves of the plant to prepare either an infusion or decoction, which was consumed for a few days until the fever subsided.

## Discussion

To our knowledge this is the first systematic ethnobotanical study done in Trinidad to determine the extent of traditional use of medicinal plants throughout several rural communities on the island. This study is significant in that it covered fifty remote communities with a sufficiently large sample size to assess the distribution of medicinal plant use for common ailments. We used the validated TRAMIL survey instrument which allowed us to determine herbal ‘home remedies’ use among these rural communities. Although we limited the number of ailments, for practical considerations, we were able to gather a wealth of information on several plants, including methods of preparation and mode and frequency of administration.

There were notable differences between our findings and that obtained in earlier surveys. Almost 40 years ago, Wong [6] identified 186 different medicinal plants from his interviews with 70 villagers at a remote community for a wide range of ailments, but only 52 of these plants had similar traditional use compared to our survey; and none were used for kidney stones or asthma. In the more extensive survey by Seaforth and colleagues across 18 rural communities [8], although 78 plants were identified, only 28 of these plants had similar traditional use compared to our survey; and none were used for kidney stones, “stoppage-of-water” or “afterbirth”. The more recent survey by Lans cited 24 plants used for “cooling/cleanser” [9], but just 11 of these plants had similar use compared to our survey.

The surveys by Clement and colleagues [10–12] focused on the complementary use of herbal remedies in patients with chronic diseases attending modern primary public healthcare facilities. Although some commonly cited plants such as *Leonotis nepetifolia*, *Zingiber officinale*, *Cymbopogon citratus* and *Aloe vera* were also found in our survey, the use of herbal remedies for culture-bound health issues such as “afterbirth”, “stoppage-of-water” and “womb infection” was notably absent. In our setting, this observation partly supports our initial assumption that there are rural–urban differences in retention of traditional knowledge and use of herbal remedies.

Although medico-cultural concepts such as “stoppage-of-water”, “womb infection” and “afterbirth” could be explained by modern medicine, and conventional therapies are available for treatment, people in remote communities may still prefer to rely on generations-old traditions as their preferred mode of treatment. The cultural interpretation of these aliments and conditions may differ from that proposed by modern medicine, and generations of anecdotal evidence would be having a significant impact on the continuation of these rural traditions.

It would be problematic to conceptualize the use of “cooling/cleanser” as prophylaxis in modern medicine, as the “hot-cold” dichotomy of health and disease has long been abandoned. But, surprisingly, “cooling” was among the most popular indications for medicinal plant use in rural Trinidad. The maintenance and restoration of the “hot-cold” balance in the body seems to resonate among rural communities across the developing world, and point to similarities in the origin of cultural beliefs regarding health and disease.

A major objective of our survey was to determine whether relevant pharmacological evidence existed that would support the traditional use of medicinal plants with significant (popular) use in our setting. A preliminary review of the literature shows that there was very sparse clinical evidence. However, we provide in the rest of this discussion the limited evidence from studies conducted in cell cultures (*in vitro*), isolated tissues (*ex vivo*) and laboratory animals (*in vivo*) which may lend support to their traditional use.

*Leonotis nepetifolia* was the most commonly cited plant being used to treat an array of conditions, including fever, common cold/cough, “cooling”, diabetes and asthma; and, the literature is sparse regarding its biological activity. In an *ex vivo* model the aqueous extract of the leaves of *L. nepetifolia* produced relaxation of pre-contracted guinea-pig tracheal rings but only at relatively high concentrations of 1000 μg/mL and this may lend support for its traditional use in the treatment of asthma [26].

*Neurolaena lobata* was the second most commonly cited plant in our survey, and was used to treat fever, the common cold/cough, diabetes and “womb infection”. The infusion, made from the leaves, is very bitter-tasting and is commonly used throughout the Caribbean and Latin America for fever, colds, malaria, ‘painful belly pains’, painful menstruation and even diabetes [27]. Toxicity studies in mice have demonstrated safety in an animal model at aqueous oral doses up to 5,000 mg/kg [28].

The literature provides pre-clinical evidence to support the biological activity for several extracts of *Neurolaena lobata* against infectious organisms, including protozoa, malaria parasite, fungi and filarial worms. The aqueous and lipophilic extracts, and isolated sesquiterpene lactones were active *in vitro* against *Plasmodium falciparium* (the parasite responsible for malaria) [29]. The methanol extract significantly reduced parasitemias in *Plasmodium berghei*-infected mice and was active against both chloroquine-susceptible and resistant *P. falciparum* strains [30]. Crude, hexane and ethanol extracts significantly inhibited both trypomastigote and epimastigote developmental forms of *Trypanosoma cruzi* [31, 32]. Extracts, fractions and isolated sesquiterpenes lactones significantly inhibited parasite growth of *Leishmania mexicana, L. cruzi and L. vaginalis* [33]. The ethanol extract exhibited a significant macrofilaricidal effect against *Brugia pahangi* (a lymphatic dwelling filiarial worm) in a concentration- and time-dependent manner [34]. Bioassay-guided fractionation of *N. lobata* demonstrated its weak to moderately active antifungal activity [35]. Additionally, the leaf extracts possessed anti-inflammatory properties in an *in vitro* LPS-stimulated monocyte model [36, 37] and analgesic properties in *in vivo* models for pain [28]. However, there is no clinical data to support the use of this plant for any of the traditional uses in Trinidad.

*Cymbopogon citratus* was the most frequently cited plant for fever, and was used to a lesser extent for the treatment of common cold/cough and as “cooling”. The essential oils of *Cymbopogon citratus* demonstrated significant analgesic and anti-inflammatory properties [38]. Other pre-clinical studies show that the aqueous extract and the essential oils of *C. citratus* possess considerable anti-inflammatory properties [39–42]. Although Carlini and colleagues [42] reported that an aqueous extract of *C. citratus* was not effective in reducing body temperature in hyperthermic mice at a dose 40 times higher than that normally used in traditional preparations, a more recent study by a group led by Ghenou [43] showed that the essential oils demonstrated strong antipyretic effects similar to a conventional analgesic agent.

*Curcuma longa* was the most commonly cited herb in our survey for the treatment of “afterbirth”. Although thousands of papers have been published regarding the biological properties of *Curcuma longa*, and its purified constituent curcumin, very little is reported regarding its use or efficacy to support its use in the postpartum period. A single study by Itthipanichong and colleagues [44] showed that curcuminoids produced a dose-dependent relaxation of oxytocin-induced contractions in isolated rat uterus. However, this *ex vivo* study does not support the traditional use of *C. longa* where it would be expected that it would cause an increase in uterine contraction to expel remnants of the “afterbirth”. However, several *in vitro* studies show that *C. longa* extracts and curcuminoids possess potent antibacterial, antifungal and antiviral properties [45] which may be beneficial during the postpartum period when there is an increased likelihood of genitourinary infections.

Our review of the literature revealed there are no reports regarding the effects of *Senna occidentalis* extracts on the uterus that could be extrapolated to its usefulness in the postnatal period. Furthermore, there is limited pre-clinical evidence regarding the antimicrobial activity of *S. occidentalis* [46]. Similarly, there were no reports regarding whether extracts of *Spondias mombin* had any effect on uterine contractility; however, there were reports that plant extracts possessed wide spectrum antibacterial properties *in vitro* [47, 48]. Although the literature does not provide evidence for the use of these plants as spasmogenic agents to expel the “afterbirth”, it may be possible that their use may be inadvertently providing antimicrobial coverage as uterine infections are common following childbirth.

The juice of *Citrus limon* was most commonly used in the management of kidney stones and several clinical studies have supported the use of lemonade to reduce the recurrence of calcium oxalate kidney stones by increasing urinary citrate levels [49–52]. Although *Gomphrena globosa* was the most commonly used plant for “stoppage-of-water” or urinary retention and kidney stones there were no pre-clinical or clinical reports in the literature to support any of these traditional uses.

Although the literature shows limited pre-clinical evidence to demonstrate pharmacological activities for some of the plants cited in our survey, this must be taken cautiously, as this level of evidence does not represent the reality in the traditional use setting. Firstly, the pre-clinical evidence comes from studies utilizing solvent extracts, fractions, or isolated compounds which are not the modality traditionally used. Secondly, the concentrations of putative components in these solvent extracts, fractions or isolated compounds used in pre-clinical experiments may be significantly higher than that which could be attained following oral administration, thus making the extrapolations to the clinical setting unjustified. However, there are a few promising examples, such as Senna (an FDA-approved non-prescription laxative) which has been clinically proven to be efficacious. A similar approach is needed to determine the clinical efficacy of other herbal remedies.

However, the identification of these medicinal plants provides a platform from which further pre-clinical and clinical studies could be formulated to determine the efficacy and safety of herbal preparations. These research efforts may provide alternative and/or complementary approaches for healthcare provision in the Caribbean and beyond.

## Conclusion

We were able to achieve our objectives and identify medicinal plants used for the most common ailments across a wide cross-section of rural communities in Trinidad. These findings add to the body of work previously done on the island, and should provide a platform for more focused surveys in the future. Our survey showed significant retention of traditional knowledge of medicinal plants in rural Trinidad. More interestingly, a large remnant of medico-cultural concepts such as “cooling/cleanser”, “afterbirth”, “stoppage-of-water” and “womb infection” persist in the rural population. Although the scientific literature show that some of the cited plants possessed antimicrobial, anti-inflammatory and related pharmacological activities in laboratory studies, these results must be taken with caution until clinical trials are conducted to establish safety and efficacy.

## Appendix 1

### TRAMIL sample survey questionnaire

Treatments used for: (local name of the problem)

Illness description (in basic English):

First treatments (the last time the problem has come out in the family):

___ traditional medicinal plants

___ healer or witch doctor

___ medical officer

Description and way of preparation of the remedy:

How did you take the remedy, in what quantity and how many times?:

Where did you find the plants?

___ yard

___ not at home

Have you already used this remedy?

___ Yes

___ No

Which results have you obtained?

What precautions should be observed during treatment?

(contraindications & side effects)

And for the children?

(contraindications and directions for use)

Available at: http://www.tramil.net/english/TramilModelo.html

## References

[1] Trinidad and Tobago 2011 Population and Housing Census Report [http://cso.planning.gov.tt/sites/default/files/content/images/census/TRINIDAD%20AND%20TOBAGO%202011%20Demographic%20Report.pdf]. Accessed 15 March 2015.

[2] Ahmad N (2011). Soils of the Caribbean.

[3] Trinidad and Tobago Diversity. [http://www.biodiversity.gov.tt/home/trinidad-a-tobago-biodiversity.html]. Accessed 15 March 2015.

[4] Simpson GE (1962). Folk medicine in Trinidad. J Am Folk.

[5] Mischel F (1959). Faith healing and medical practice in the southern Caribbean. Southwest J Anthropol.

[6] Wong W (1976). Some folk medicinal plants from Trinidad. Econ Bot.

[7] Clement Y, Sutherland P, Moodley P, Chevannes B (2014). Herbal medicine practices in the Caribbean. Caribbean Healing Traditions – Implications for health and mental health.

[8] Seaforth CE, Adams CD, Sylvester Y (1983). A guide to the medicinal plants of Trinidad & Tobago.

[9] Lans C (2006). Ethnomedicines used in Trinidad and Tobago for urinary problems and diabetes mellitus. J Ethnobio Ethnomed.

[10] Clement YN, Williams AF, Aranda D, Chase R, Watson N, Mohammed R (2005). Medicinal herb use among asthmatic patients attending a specialty care facility in Trinidad. BMC Complement Altern Med.

[11] Clement YN, Morton-Gittens J, Basdeo L, Blades A, Francis MJ, Gomes N (2007). Perceived efficacy of herbal remedies by users accessing primary healthcare in Trinidad. BMC Complement Altern Med.

[12] Clement YN (2009). Herbal self-medication at primary health care facilities in Trinidad. J Altern Complement Med.

[13] Pardo-de-Santayana M, Pieroni A, Puri RK (2013). Ethnobotany in the New Europe: People Health and Wild Pland Resources.

[14] Chew-Chung WA (1980). Many paths to health: A study of 2,000 rural and urban Taiwan families. Am J Chin Med.

[15] Adams J, Sibbritt D, Broom A, Loxton D, Pirotta M, Humphreys J (2011). A comparison of complementary and alternative medicine users and use across geographical areas: A national survey of 1,427 women. BMC Complement Altern Med.

[16] Robineau L, Saejarto DD, Balick MJ, Elizabetski E, Laird SA (1986). TRAMIL: a research project on the medicinal resources of the Caribbean. Medicinal Resources of the Tropical Forest (Biodiversity and its importance to Human Health).

[17] Elections and Boundaries Commission, Government of Trinidad and Tobago [http://www.ebctt.com/index.php]. Accessed 15 March 2015.

[18] van Andel T, de Boer HJ, Barnes J, Vandebroek I (2014). Medicinal plants used for menstrual disorders in Latin America, the Caribbean, sub-Saharan Africa, south and Southeast Asia and their uterine properties: A review. J Ethnopharmacol.

[19] de Boer HJ, Cotingting C (2014). Medicinal plants for women’s healthcare in southeast Asia: A meta-analysis of their traditional use, chemical constituents, and pharmacology. J Ethnopharmacol.

[20] Zumsteg IS, Weckerle CS (2007). Bakera, a herbal steam bath for postnatal care in Minahasa (Indonesia): Documentation of the plants used and assessment of the method. J Ethnopharmacol.

[21] Kaingu CK, Oduma JA, Kanui TI (2011). Practices of traditional birth attendants in Machakos district, Kenya. J Ethnophramacol.

[22] Foster GM (1979). Humoral traces in United States folk medicine. Med Anthropol Newsl.

[23] Foster GM (1988). The validating role of humoral theory in traditional Spanish-American therapeutics. Am Anthropol.

[24] Jackson WA (2001). A short guide to humoral medicine. TiPs.

[25] Schoental R (1957). Herbal medicines and disease. J Trop Ped.

[26] Calixto JB, Yunes RA, Rae GA (1991). Effect of crude extracts from *Leonotis nepetaefolia* (Labiatae) on rat and guinea-pig smooth muscle and rat cardiac muscle. J Pharm Pharmacol.

[27] Hawthorne WD, Jules D, Marcelle G (2004). Caribbean Spice Islands Plants.

[28] Gracioso JS, Paulo MQ, Hiruma Lima CA, Souza Brito AR (1998). Antinociceptive effect in mice of a hydroalcoholic extract of *Neurolaena lobata* (L.) R. Br. and its organic fractions. J Pharm Pharmacol.

[29] François G, Passreiter CM, Woerdenbag HJ, Van Looveren M (1996). Antiplasmodial activities and cytotoxic effects of aqueous extracts and sesquiterpene lactones from *Neurolaena lobata*. Planta Med.

[30] Franssen FF, Smeijsters LJ, Berger I, Medinilla Aldana BE (1997). *In vivo* and *in vitro* antiplasmodial activities of some plants traditionally used in Guatemala against malaria. Antimicrob Agents Chemother.

[31] Berger I, Barrientos AC, Cáceres A, Hernández M, Rastrelli L, Passreiter CM (1998). Plants used in Guatemala for the treatment of protozoal infections: II. Activity of extracts and fractions of five Guatemalan plants against *Trypanosoma cruzi*. J Ethnopharmacol.

[32] Cáceres A, López B, González S, Berger I, Tada I, Maki J (1998). Plants used in Guatemala for the treatment of protozoal infections. I. Screening of activity to bacteria, fungi and American trypanosomes of 13 native plants. J Ethnopharmacol.

[33] Berger I, Passreiter CM, Cáceres A, Kubelka W (2001). Antiprotozoal activity of *Neurolaena lobata*. Phytother Res.

[34] Fujimaki Y, Kamachi T, Yanagi T, Cáceres A, Maki J, Aoki Y (2005). Macrofilaricidal and microfilaricidal effects of *Neurolaena lobata*, a Guatemalan medicinal plant, on *Brugia pahangi*. J Helminthol.

[35] Lentz DL, Clark AM, Hufford CD, Meurer-Grimes B, Passreiter CM, Cordero J (1998). Antimicrobial properties of Honduran medicinal plants. J Ethnopharmacol.

[36] Lajter I, Vasas A, Béni Z, Forgo P, Binder M, Bochkov V (2014). Sesquiterpenes from *Neurolaena lobata* and their antiproliferative and anti-inflammatory activities. J Nat Prod.

[37] Walshe-Roussel B, Choueiri C, Saleem A, Asim M, Caal F, Cal V (2013). Potent anti-inflammatory activity of sesquiterpene lactones from *Neurolaena lobata* (L.) R. Br. ex Cass., a Q'eqchi' Maya traditional medicine. Phytochemistry.

[38] Brito RG, Guimarães AG, Quintans JS, Santos MR, De Sousa DP, Badaue-Passos D (2012). Citronellol, a monoterpene alcohol, reduces nociceptive and inflammatory activities in rodents. J Nat Med.

[39] Sforcin JM, Amaral JT, Fernández A, Sousa JP, Bastos JK (2009). Lemongrass effects on IL-1beta and IL-6 production by macrophages. Nat Prod Res.

[40] Figueirinha A, Cruz MT, Francisco V, Lopes MC, Batista MT (2010). Anti-inflammatory activity of *Cymbopogon citratus* leaf infusion in lipopolysaccharide-stimulated dendritic cells: contribution of the polyphenols. J Med Food.

[41] Mitoshi M, Kuriyama I, Nakayama H, Miyazato H, Sugimoto K, Kobayashi Y (2014). Suppression of allergic and inflammatory responses by essential oils derived from herbal plants and citrus fruits. Int J Mol Med.

[42] Carlini EA, de DP CJ, Silva-Filho AR, da Silveira-Filho NG, Frochtengarten ML, Bueno OF (1986). Pharmacology of lemongrass (*Cymbopogon citratus* Stapf). I. Effects of teas prepared from the leaves on laboratory animals. J Ethnopharmacol.

[43] Ghenou JD, Ahounou JF, Akakpo HB, Laleye A, Yayi E, Gbaguidi F (2013). Phytochemical composition of *Cymbopogon citratus* and *Eucalyptus citriodora* essential oils and their anti-inflammatory and analgesic properties on Wistar rats. Mol Biol Rep.

[44] Itthipanichpong C, Ruangrungsi N, Kemsri W, Sawasdipanich A (2003). Antispasmodic effects of curcuminoids on isolated guinea-pig ileum and rat uterus. J Med Assoc Thai.

[45] Moghadamtousi SZ, Kadir HA, Hassandarvish P, Tajik H, Abubakar S, Zandi K (2014). A review on antibacterial, antiviral, and antifungal activity of curcumin. Biomed Res Int.

[46] Samy RP, Ignacimuthu S (2000). Antibacterial activity of some folklore medicinal plants used by tribals in Western Ghats of India. J Ethnopharmacol.

[47] Abo KA, Ogunleye VO, Ashidi JS (1999). Antimicrobial potential of *Spondias mombin*, *Croton zambesicus* and *Zygotritonia crocea*. Phytother Res.

[48] Corthout J, Pieters L, Claeys M, Geerts S, Vanden Berghe D, Vlietinck A (1994). Antibacterial and molluscicidal phenolic acids from *Spondias mombin*. Planta Med.

[49] Tosukhowong P, Yachantha C, Sasivongsbhakdi T, Ratchanon S, Chaisawasdi S, Boonla C (2008). Citraturic, alkalinizing and antioxidative effects of limeade-based regimen in nephrolithiasis patients. Urol Res.

[50] Penniston KL, Steele TH, Nakada SY (2007). Lemonade therapy increases urinary citrate and urine volumes in patients with recurrent calcium oxalate stone formation. Urology.

[51] Kang DE, Sur RL, Haleblian GE, Fitzsimons NJ, Borawski KM, Preminger GM (2007). Long-term lemonade based dietary manipulation in patients with hypocitraturic nephrolithiasis. J Urol.

[52] Seltzer MA, Low RK, McDonald M, Shami GS, Stoller ML (1996). Dietary manipulation with lemonade to treat hypocitraturic calcium nephrolithiasis. J Urol.

